# Factors associated with tuberculosis patient delay: a meta-analysis

**DOI:** 10.3389/fpubh.2026.1832410

**Published:** 2026-07-14

**Authors:** Yi Liu, Yuansi Huang, Chunli Fan

**Affiliations:** Nursing Department, Chenzhou Municipal Hospital of Traditional Chinese Medicine, Chenzhou, Hunan, China

**Keywords:** adenomyosis, laparoscopic adenomyomectomy, nomogram, risk prediction, symptomatic recurrence

## Abstract

**Background:**

Tuberculosis (TB) is among the top 10 causes of global death, and patient delay is a key factor causing higher mortality. This study intends to analyze the associated factors with TB patient delay.

**Methods:**

Embase, Cochrane Library, PubMed, and Web of Science were systematically searched for online available studies and unpublished sources from inception to 2025. STATA15.1 was adopted for data analyses, heterogeneity by inverse variance (*I*^2^) and Cochran’s *Q*-tests, and publication bias by Egger’s tests. The Newcastle–Ottawa Scale was utilized for assessing the quality of cohort and case–control studies, and the Agency for Healthcare Research and Quality criteria for cross-sectional studies.

**Results:**

Forty-two studies were included, involving 492,448 patients. The meta-analysis showed that occupation (OR 1.20, 95% CI 1.10–1.30, *p* < 0.001), educational level (OR 1.37, 95% CI 1.007–1.76, *p* = 0.012), place of residence (OR 1.66, 95% CI 1.44–1.92, *p* < 0.001), TB classification (OR 1.31, 95% CI 1.15–1.49, *p* < 0.001), diabetes (OR 0.62, 95% CI 0.47–0.81, *p* < 0.001), HIV infection (OR 0.80, 95% CI 0.72–0.89, *p* = 0.032), drinking (OR 1.43, 95% CI 1.29–1.59, *p* < 0.001), TB knowledge (OR 2.09, 95% CI 1.61–2.71, *p* < 0.001), stigma (OR 1.87, 95% CI 1.48–2.36, *p* < 0.001), and time of arrival at health services (OR 1.34, 95% CI 1.03–1.74, *p* = 0.03) were closely associated with patient delay. Age, sex, marital status, BMI, insurance, smoking, knowing someone with TB, and distance to health services had no associations with patient delay.

**Conclusion:**

Patient delay not only affects the treatment and prognosis but also worsens the burden on the family and society. The rate of patient delay can be reduced by strengthening health education, establishing a perfect service system, and implementing early screening.

## 1 Introduction

Tuberculosis (TB) resulting from *Mycobacterium tuberculosis* infection is one of the top 10 causes of global death (1). As reported by the WHO (2023), TB has a prevalence of approximately 133/100,000 (2), but only 47% of patients promptly seek medical care, whereas up to 42% of them do not come to hospitals until related symptoms have persisted ≥1 month (3). Patient delay refers to an interval of over 2 weeks from the emergence of clinical symptoms to the first medical visit by TB patients (4). Only patients with obvious or severe TB symptoms will actively seek medical care in China (5), Mongolia (6), Portugal (7), India (8), and Ethiopia (9), missing the optimal window for treatment, and the median number of patient delays is on the rise (10). It is estimated that an untreated smear-positive TB can cause 10–15 contact infections per year, with approximately 20 infections in the disease history until death (11). Patient delay is therefore a key factor contributing to drug resistance, severe conditions at diagnosis, poor response to treatment, sequelae, and increases in overall mortality in TB patients.

The factors influencing TB patient delay have been explored. Helfinstein et al. argued that patient delay is related to sex, age, educational level, and disease knowledge (12). Ribeiro RM et al. argued that patient delay has associations with occupation, income, place of residence, smoking, drinking, or other drugs, but is less related to sex and age (13). Other scholars believe that besides patient factors, insufficient social support, inadequate health services, and distance from the patient’s residence to health services all influence patient delay (14–17). Despite preliminary studies, the conclusions remain inconsistent. Therefore, this meta-analysis intends to analyze the influencing factors for TB patient delay and identify relevant risk factors, thereby providing some reference for future interventions and policy development.

## 2 Methods

This meta-analysis adhered to the PRISMA (18) and was registered with PROSPERO (CRD42024540454).

### 2.1 Search strategy

We searched PubMed, Web of Science, Embase, and Cochrane Library with the MeSH terms “Tuberculosis, Pulmonary,” “lung tuberculosis,” and “Delay” from inception to December 31, 2025. Due to the word limit, the search formula is provided in Appendix 1. We also manually checked the reference lists of the included studies to ensure no omissions.

### 2.2 Eligibility criteria

Inclusion criteria: (1) Population: TB patients. (2) Exposure: patient delay and associated factors. (3) Comparator: TB patients without delay. (4) Outcome: Rate of TB patient delay. (5) Study type: cohort, cross-sectional, and case–control studies. Exclusion criteria: (1) Cellular or animal experiments, reviews, experiment plans, case reports, letters, conference papers, and editorials. (2) Data missing or major errors. (3) Duplicate publications. (4) Unavailable full text.

### 2.3 Data extraction

After duplicate removal by EndNote, two investigators (Liu and Huan) checked the title and abstract according to the eligibility criteria, and reviewed the full text then. They settled disagreements by discussion or consultation with a third investigator (Fan). The two investigators extracted data (first author, country, publication year, sample size, study type, sex, age, and number of patient delays) using Excel2016.

### 2.4 Quality assessment

Liu and Huan were independently responsible for quality assessment, and Fan for the resolution of the disagreement. The Newcastle–Ottawa Scale (19, 20) was utilized for assessing cohort and case–control studies from selection, comparability, and exposure (case–control) or outcome (cohort). For each item in the Selection and Exposure/Outcome, a study may receive up to one star. For Comparability, a maximum of two stars may be granted. 0–3, 4–6, and 7–9 points corresponded to low, medium, and high quality, respectively. In addition, the cross-sectional studies were assessed using an 11-item checklist (source of information, eligibility criteria, the period and continuity for identifying patients, masking, confounder and missing data, completeness, quality assessment, and response rate) provided by the Agency for Healthcare Research and Quality (21). The answer “Unclear” or “No” was given zero points, and “Yes” was given one point. 0–3, 4–7, and 8–11 points corresponded to low, medium, and high quality, respectively.

### 2.5 Data analysis

STATA15.1 (StataCorp LP, College Station, Texas, USA) was employed. Risk factors were described by odds ratio (OR, the ratio of the odds of exposure among cases to the odds of exposure among controls; primarily used for case–control studies and logistic regression to measure the strength of the association between exposure factors and diseases) with 95% CI (a numerical interval calculated based on samples, within which the overall actual values typically fall; the 95% CI is the most commonly used in practice). Statistical significance was set at *p* < 0.05 (two-sided). Heterogeneity was measured by inverse variance (*I*^2^; small: *I*^2^ ≤ 25%, medium: 25% < *I*^2^ ≤ 50%, large: 50% < *I*^2^ ≤ 75%, or very large: *I*^2^ > 75%) and Cochran’s *Q*-test. We employed a fixed-effects model in case of acceptable heterogeneity (*I*^2^ < 50%, *p* > 0.1). A random-effects model was employed to account for significant heterogeneity (*I*^2^ > 50%, *p* < 0.1). We evaluated risk factors by leave-one-out sensitivity analyses and publication bias by Egger’s tests. This is a linear regression test used in meta-analyses to quantitatively measure publication bias. By regression analyses of the effect size and standard error, whether a result bias is present in small-sample studies is determined to assess the presence or absence of publication bias, with *p* > 0.05 as insignificant bias. The influence of publication bias was evaluated by nonparametric clipping. Publication bias refers to the condition where positive, statistically significant results are more likely to be published, while negative, non-statistically significant results are less likely to be published or are omitted. This leads to an incomplete inclusion of studies in the meta-analysis, ultimately overestimating the true effect.

## 3 Results

### 3.1 Search results

**Figure 1:**
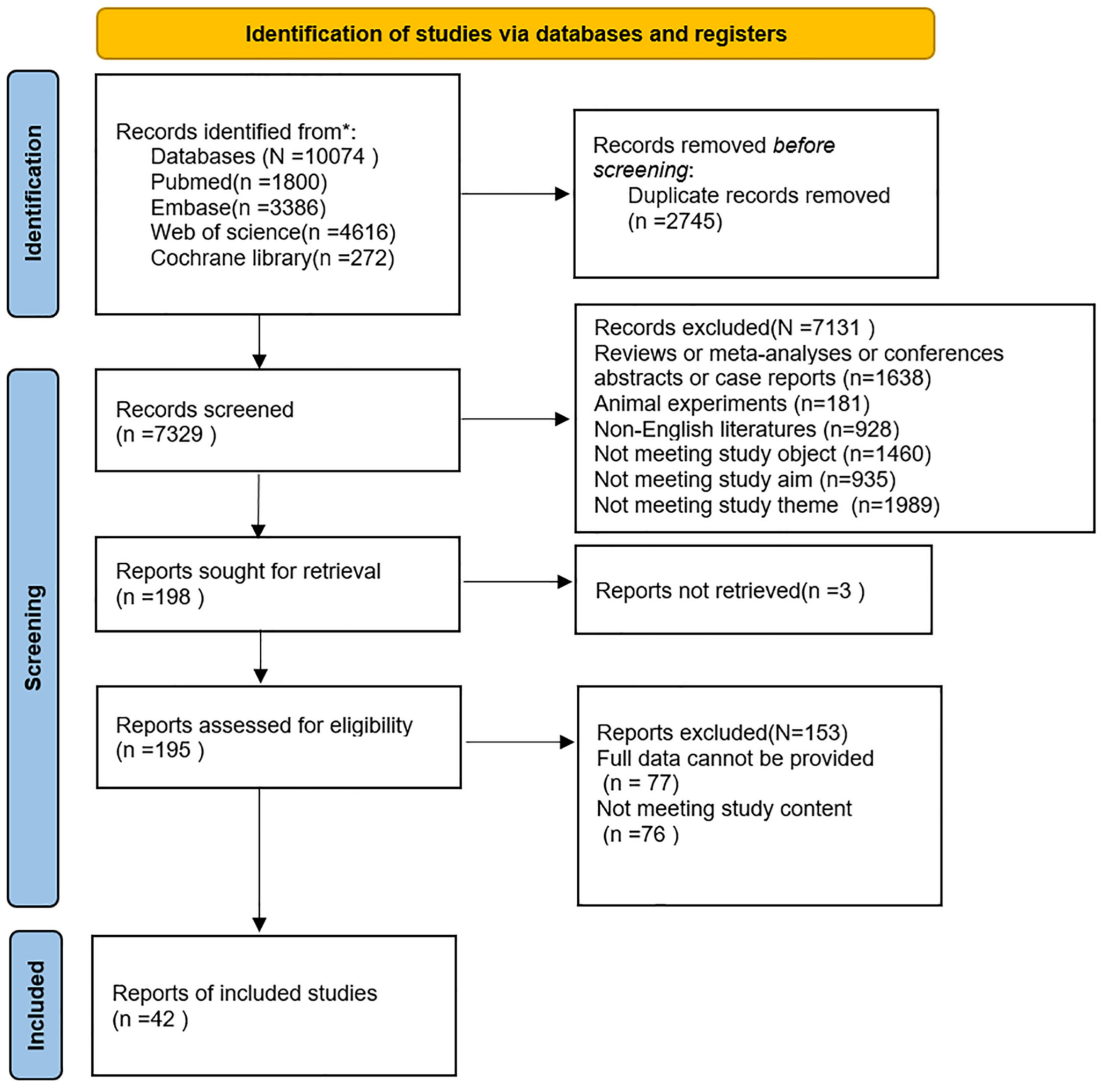
PRISMA flowchart.

We obtained 10,074 studies initially, with 2,745 excluded as duplicate publications and 7,329 excluded after reading titles and abstracts. Then the remainder was reviewed for full text following the eligibility criteria. Finally, 42 studies (5–9, 22–58) were included (Figure 1).

### 3.2 Study characteristics and quality

The included studies (7 cohort, 2 case–control, and 33 cross-sectional studies) were from 18 countries (India, China, Tajikistan, Ethiopia, Portugal, Korea, Malaysia, Mongolia, Iran, UK, Malawi, Zimbabwe, Nigeria, Mozambique, Afghanistan, Indonesia, Tanzania, and Mulago), involving 492,448 patients including 340,293 males and 152,155 females (Table 1). All studies had quality scores >7, suggesting high quality (Tables 2–4).

**Table 1:** Basic characteristics.

Author	Year	Country	Study type	Sample size	Age	Sex (M/F)	Number of patient delays
Routray et al. (22)	2025	India	Cross-sectional	420	40.85 ± 13.56	282/138	383
Malik et al. (23)	2025	India	Cross-sectional	416	40.9 ± 16.33	259/157	151
Feng et al. (24)	2025	China	Cohort	79,877	41–50	54,130/25,747	22,283
Duan et al. (25)	2025	China	Cohort	134,975	44–59	95,410/39,565	91,595
Zhu et al. (26)	2024	China	Cohort	17,078	20–39	12,786/4,292	11,573
Zhou et al. (27)	2024	China	Cohort	1,274	13	624/650	632
Sharifov et al. (28)	2024	Tajikistan	Cohort	472	33	233/239	390
Getiye et al. (29)	2024	Ethiopia	Cross-sectional	420	35.89 ± 15.82	207/213	262
Ge et al. (30)	2024	China	Cross-sectional	5,282	10–24	3,256/2,026	2,580
Animut et al. (31)	2024	India	Cross-sectional	420	40.85 ± 13.56	282/138	383
Yasobant et al. (32)	2023	India	Cross-sectional	990	18–40	586/404	419
Xie et al. (33)	2023	China	Cross-sectional	3,756	39–50	2,695/1,061	1,459
Liu et al. (34)	2023	China	Cross-sectional	3,442	15–59	2,343/1,099	1,594
Guo et al. (5)	2023	China	Cross-sectional	3,190	52 ± 33.67	2,258/932	2,268
Ereso et al. (9)	2023	Ethiopia	Cross-sectional	1,161	32.2 ± 14.41	594/567	539
deMorais et al. (7)	2023	Portugal	Cross-sectional	75	50	57/18	42
Zhu et al. (35)	2022	China	Cohort	208,822	0–45	146,538/62,284	120,926
Ko et al. (36)	2022	Korea	Case–control	6,593	61.2 ± 18.8	3,528/3,065	2,442
Cheong et al. (37)	2022	Malaysia	Cross-sectional	732	40	391/241	360
Batbayar et al. (6)	2022	Mongolia	Cross-sectional	3,267	32	1,836/1,431	3,267
Balasubramnian et al. (8)	2022	India	Cross-sectional	229	51.21 ± 13.79	194/35	98
Moosazadeh et al. (38)	2021	Iran	Cross-sectional	173	55.6	88/85	86
Arja et al. (39)	2021	Ethiopia	Cross-sectional	255	25	149/106	145
Datiko et al. (40)	2020	Ethiopia	Cross-sectional	844	34 ± 13.8	488/356	575
Wondawek et al. (41)	2019	Ethiopia	Cross-sectional	598	25–34	271/327	462
Evenden et al. (42)	2019	Britain	Cohort	7,216	38	4,580/2,636	4,539
Seid et al. (43)	2018	Ethiopia	Cross-sectional	382	37.14	201/181	157
Takarinda et al. (44)	2015	Zimbabwe	Cross-sectional	383	34	211/172	184
Makwakwa et al. (45)	2014	Malawi	Cross-sectional	588	25–34	388/200	588
Gebeyehu et al. (46)	2014	Ethiopia	Cross-sectional	360	20–39	214/146	360
Biya et al. (47)	2014	Nigeria	Cross-sectional	160	32.8 ± 9	99/61	67
Xu et al. (48)	2013	China	Case–control	4,677	15–30	2,979/1,698	1,698
Saifodine et al. (49)	2013	Mozambique	Cross-sectional	622	32	350/272	250
Tobe et al. (50)	2013	China	Cross-sectional	314	32	199/155	128
Sabawoon et al. (51)	2011	Afghanistan	Cross-sectional	259	15	110/149	259
Lock et al. (52)	2011	Indonesia	Cross-sectional	194	39 ± 14.1	104/90	194
Ahmad et al. (53)	2011	Indonesia	Cross-sectional	253	25–34	153/100	253
Ngadaya et al. (54)	2009	Tanzania	Cross-sectional	226	18–40	137/69	79
Mesfin et al. (55)	2009	Ethiopia	Cross-sectional	924	34	511/413	493
Kiwuwa et al. (56)	2005	Mulago	Cross-sectional	231	30.7 ± 9.4	132/99	205
Lewis et al. (57)	2003	Ethiopia	Cross-sectional	198	15–35	86/111	197
Demissie et al. (58)	2003	Ethiopia	Cross-sectional	700	31	372/328	408

**Table 2:** Agency for healthcare research and quality checklist for 33 cross-sectional studies.

Study	Define the information source (record review, survey)	Eligibility criteria for exposed and unexposed subjects (cases and controls) or previous publications	Period for identifying patients	Whether subjects were consecutive if not population-based	Evaluators of subjective components of study were masked to other aspects of the status of the participants	Assessments for quality assurance purposes (e.g., test/retest of primary outcome)	Any patient exclusion from analysis	How confounders were assessed and/or controlled	Handling of missing data, if applicable	Response rates and completeness of data collection	What follow-up, if any, was expected and the percentage of patients with incomplete data or follow-up	Total score
Routray et al. (22)	1	1	1	1	1	1	1	1	1	1	1	11
Malik et al. (23)	1	1	1	1	1	1	1	1	1	1	1	11
Ge et al. (30)	1	0	1	1	1	0	0	1	0	1	1	7
Animut et al. (31)	1	0	1	1	1	0	1	1	1	1	1	9
Yasobant et al. (32)	1	0	1	1	1	0	0	1	0	1	1	7
Xie et al. (33)	1	0	1	0	1	1	1	1	1	1	1	9
Liu et al. (34)	1	0	1	0	1	1	0	1	1	1	1	8
Guo et al. (5)	1	0	1	1	1	1	0	1	1	1	1	9
Ereso et al. (9)	1	1	1	1	1	1	1	1	1	1	1	11
deMorais et al. (7)	1	1	1	0	1	0	0	1	1	1	1	8
Cheong et al. (37)	1	1	1	0	1	1	1	1	1	1	1	10
Batbayar et al. (6)	1	1	1	0	1	1	1	1	1	1	1	10
Balasubramnian et al. (8)	1	1	1	1	1	1	1	1	1	1	1	11
Moosazadeh et al. (38)	1	1	1	0	1	1	1	1	1	1	1	10
Arja et al. (39)	1	1	1	1	1	0	0	1	1	1	1	9
Datiko et al. (40)	1	0	1	1	1	1	1	1	0	1	1	9
Wondawek et al. (41)	1	0	1	1	1	0	1	1	0	1	1	8
Seid et al. (43)	1	1	1	1	1	1	1	1	1	0	1	10
Takarinda et al. (44)	1	1	1	1	1	1	1	1	1	0	1	10
Makwakwa et al. (45)	1	1	1	0	1	1	1	1	0	1	1	9
Gebeyehu et al. (46)	1	1	1	1	1	1	1	1	0	0	1	9
Biya et al. (47)	1	0	1	1	1	1	1	1	0	0	1	9
Saifodine et al. (49)	1	0	1	0	1	1	1	1	0	1	1	8
Tobe (50)	1	0	1	0	1	1	1	1	0	1	1	8
Sabawoon et al. (51)	1	0	1	0	1	1	1	1	0	0	1	7
Lock et al. (52)	1	1	1	1	1	1	1	1	0	1	1	10
Ahmad et al. (53)	1	1	1	1	1	0	0	1	0	0	1	7
Ngadaya et al. (54)	1	1	1	1	1	1	0	1	0	1	1	9
Mesfin et al. (55)	1	1	1	1	1	0	1	1	0	1	1	9
Kiwuwa et al. (56)	1	1	1	1	1	1	1	1	0	0	1	9
Lewis et al. (57)	1	1	1	1	1	0	1	1	1	1	1	10
Demissie et al. (58)	1	1	1	1	1	1	1	1	0	0	1	9

**Table 3:** Quality assessment results (NOS) for 7 cohort studies.

Study	Selection				Comparability	Outcome			Quality scores
	Representative exposed cohort	Nonexposed cohort selection	Exposure ascertainment	Outcome not present at start	Cohort comparability	Outcome assessment	Follow-up long enough for outcomes	Adequacy of follow up	
Feng et al. (24)	1	1	1	1	1	1	1	1	8
Duan et al. (25)	1	1	1	1	1	1	1	1	8
Zhu et al. (26)	1	1	1	1	1	1	1	1	8
Zhou et al. (27)	1	1	1	1	1	1	1	1	8
Sharifov et al. (28)	1	1	1	1	1	1	1	1	8
Zhu et al. (35)	1	1	1	1	1	1	1	1	8
Evenden et al. (42)	1	1	1	1	1	1	1	1	8

**Table 4:** Quality assessment results (NOS) for 2 case–control studies.

Study	Selection				Comparability	Outcome			Quality scores
	Adequate case definition?	Representative cases	Control selection	Control definition	Cohort comparability based on design or analysis	Exposure ascertainment	Same method of case and control ascertainment	Non-response rate	
Ko et al. (36)	1	1	1	1	2	1	1	-	8
Xu et al.(48)	1	1	1	1	1	1	1	1	8

### 3.3 Meta-analysis results

The risk factors for patient delay were classified into personal factors (Figures 2–4), external factors (Figures 5–7), and disease-related factors (Figures 8–10).

**Figure 2:**
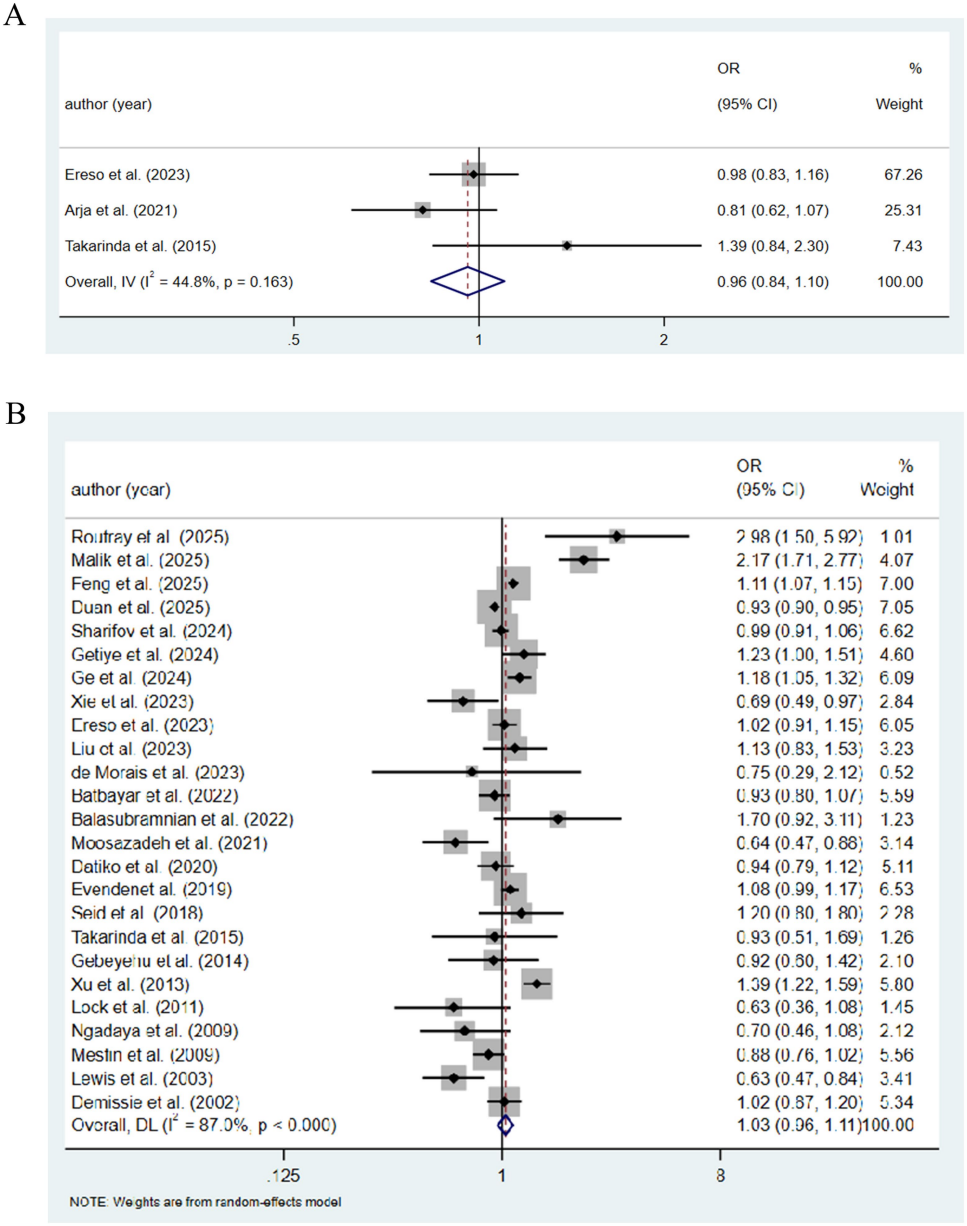
**(A)** Association of age with patient delay; **(B)** Association of sex with patient delay.

**Figure 3:**
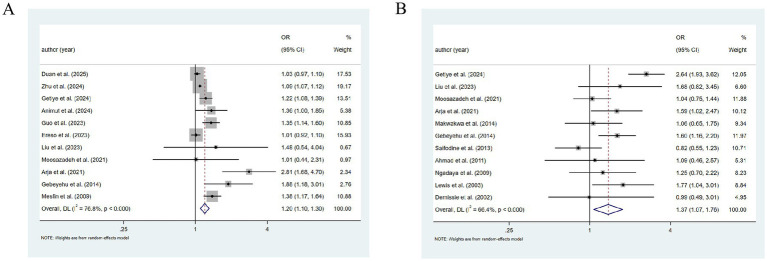
**(A)** Association of occupation with patient delay; **(B)** Association of educational level with patient delay.

**Figure 4:**
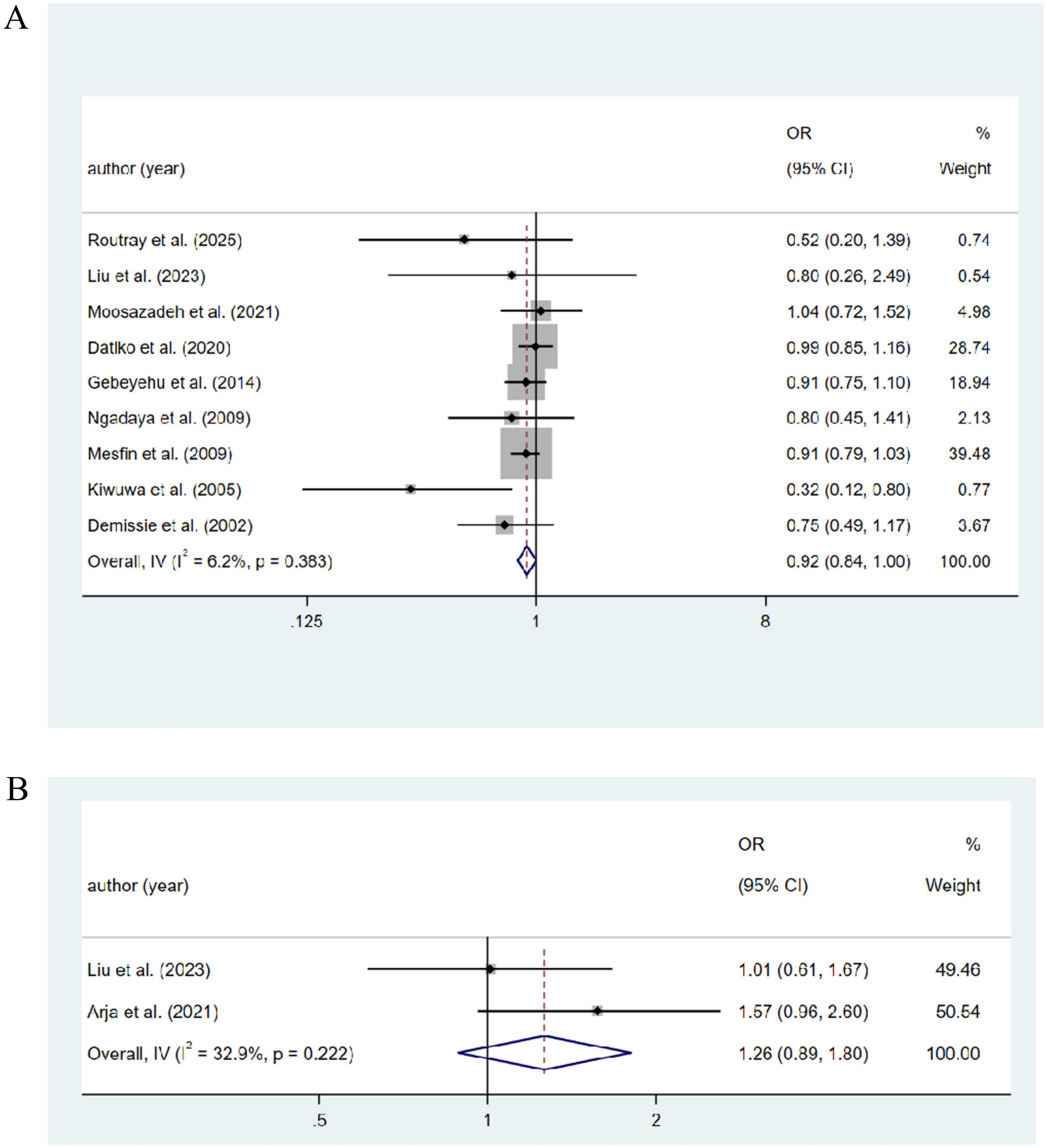
**(A)** Association of marital status with patient delay; **(B)** Association of BMI with patient delay.

**Figure 5:**
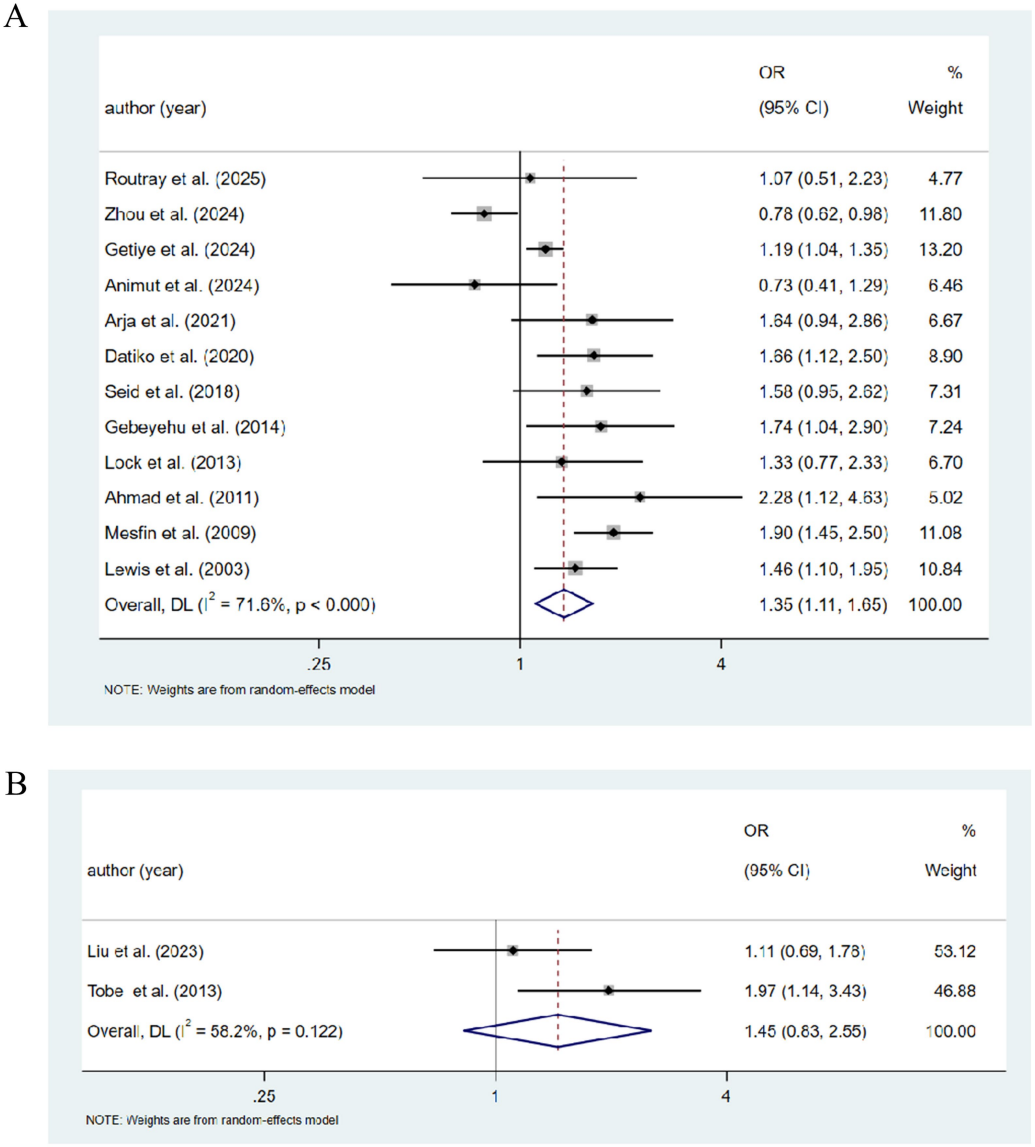
**(A)** Association of place of residence with patient delay; **(B)** Association of insurance with patient delay.

**Figure 6:**
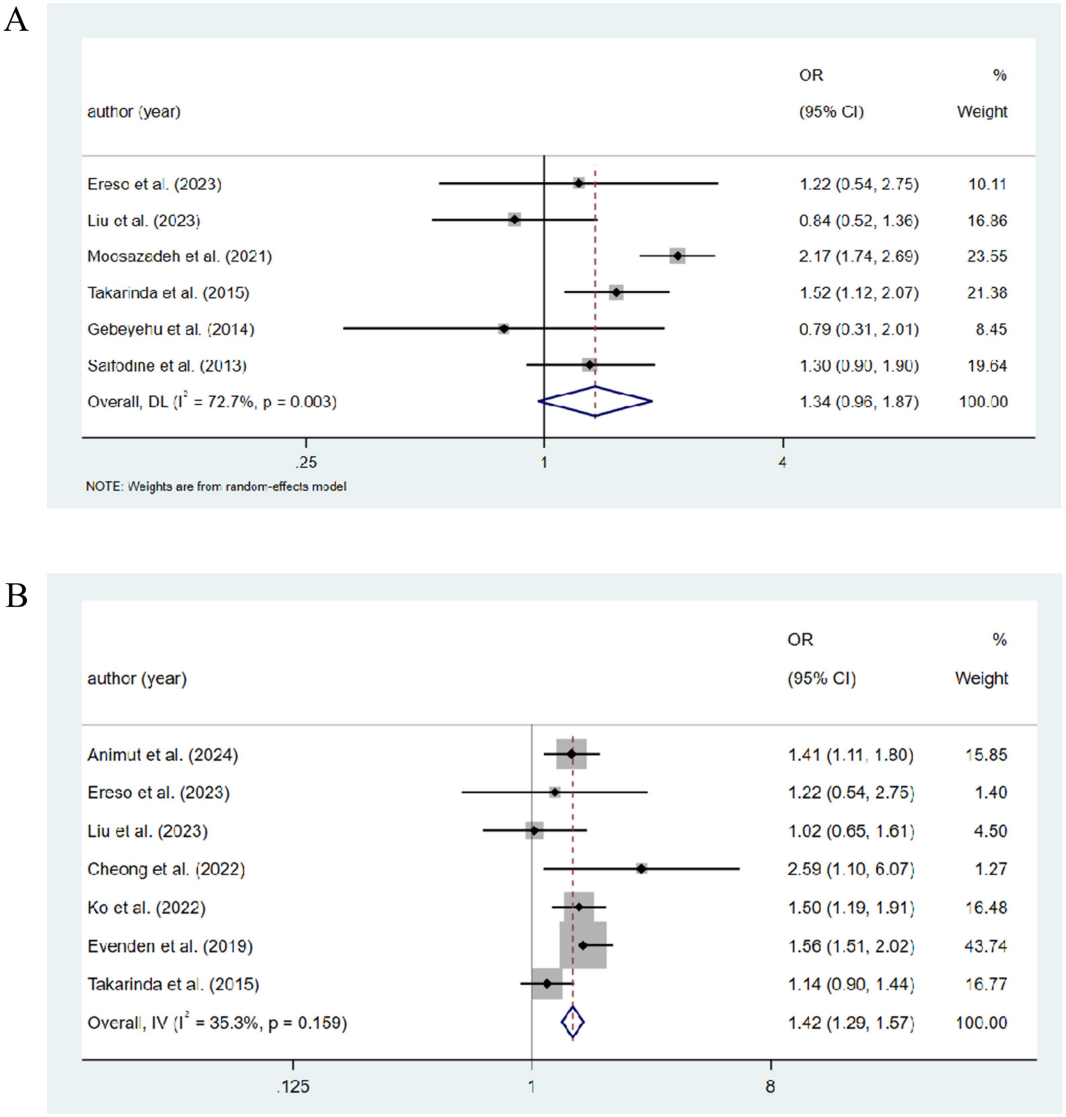
**(A)** Association of smoking with patient delay; **(B)** Association of drinking with patient delay.

**Figure 7:**
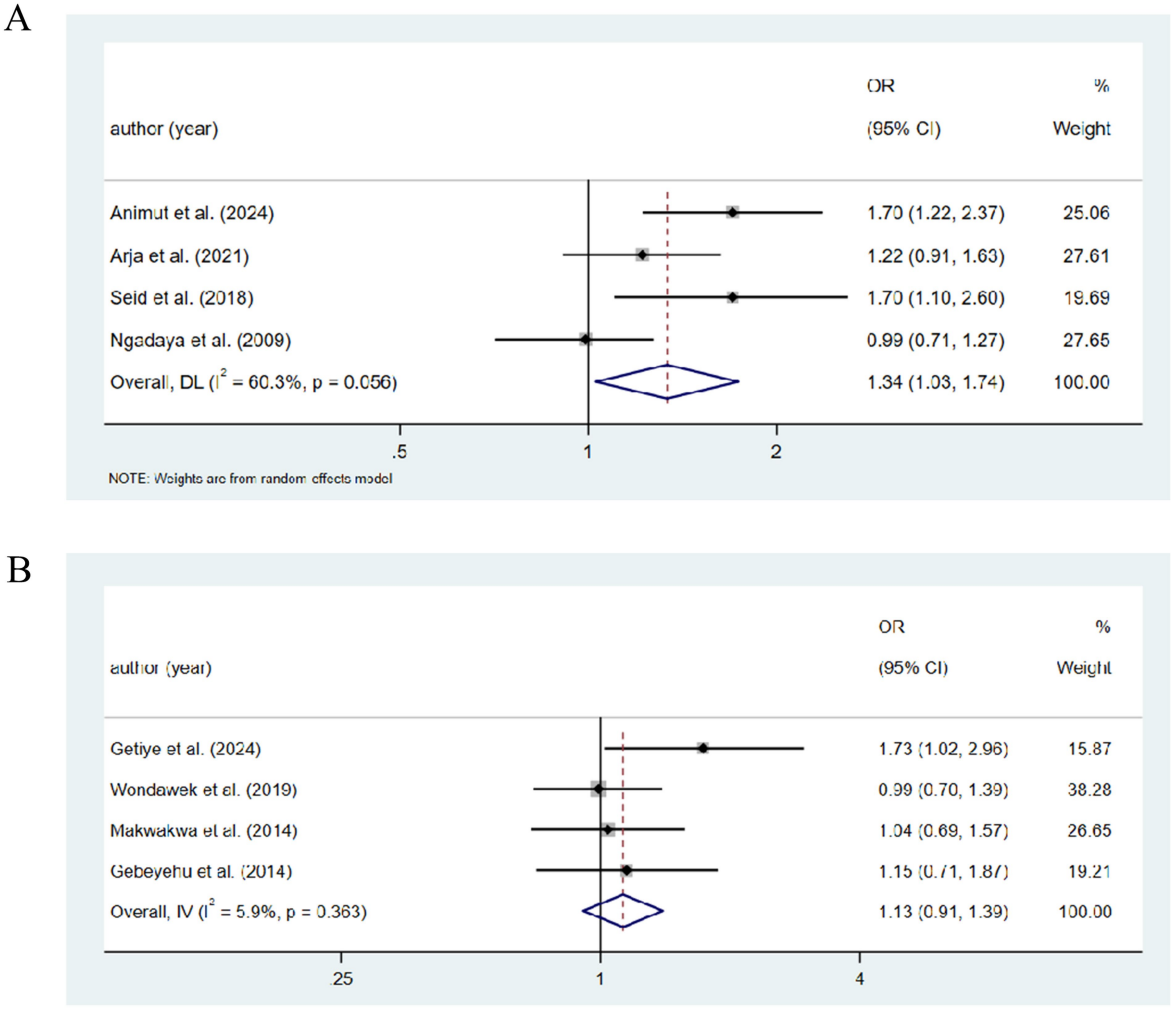
**(A)** Association of time of arrival at health services with patient delay; **(B)** Association of distance to health services with patient delay.

**Figure 8:**
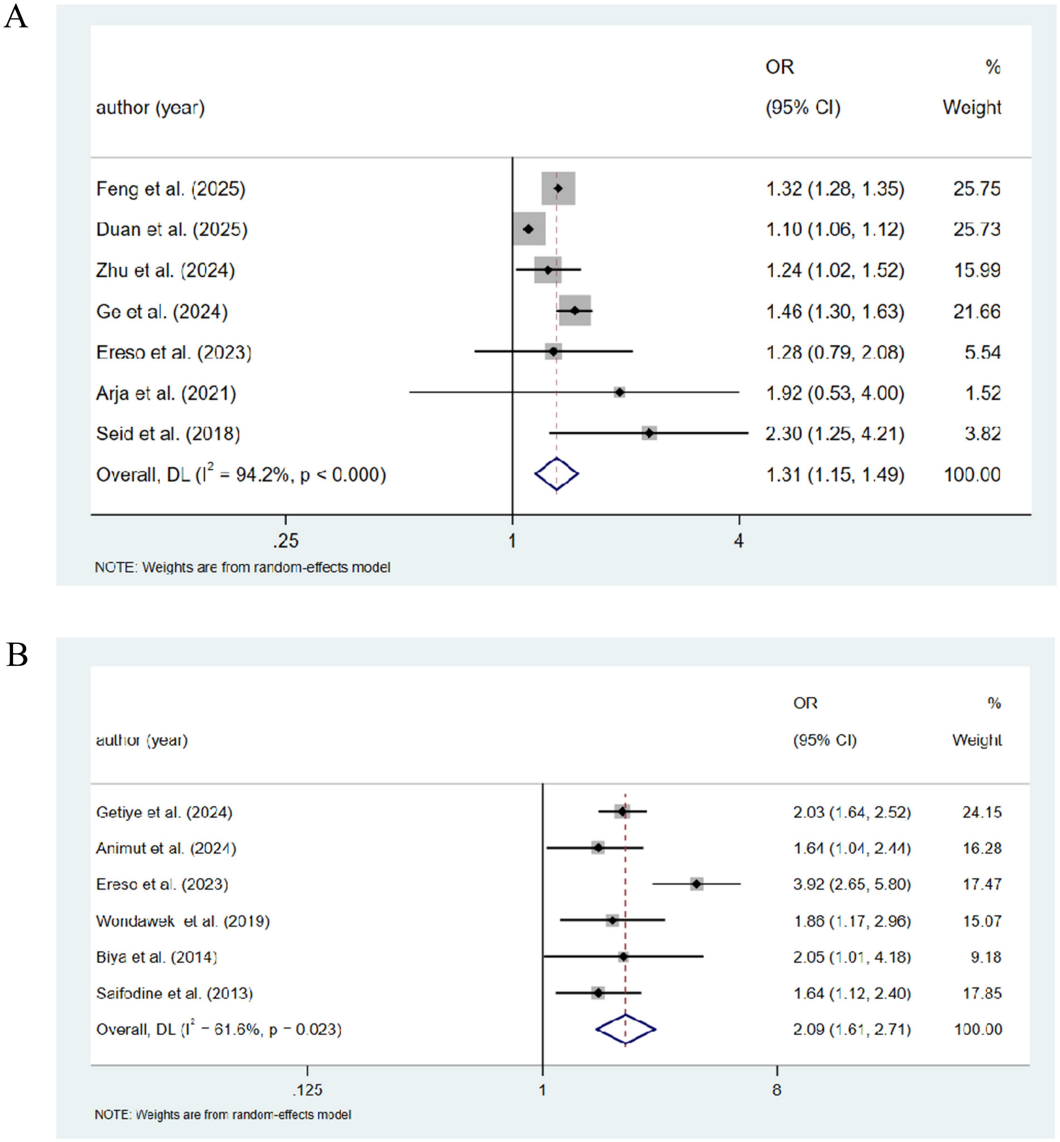
**(A)** Association of TB classification with patient delay; **(B)** Association of TB knowledge with patient delay.

**Figure 9:**
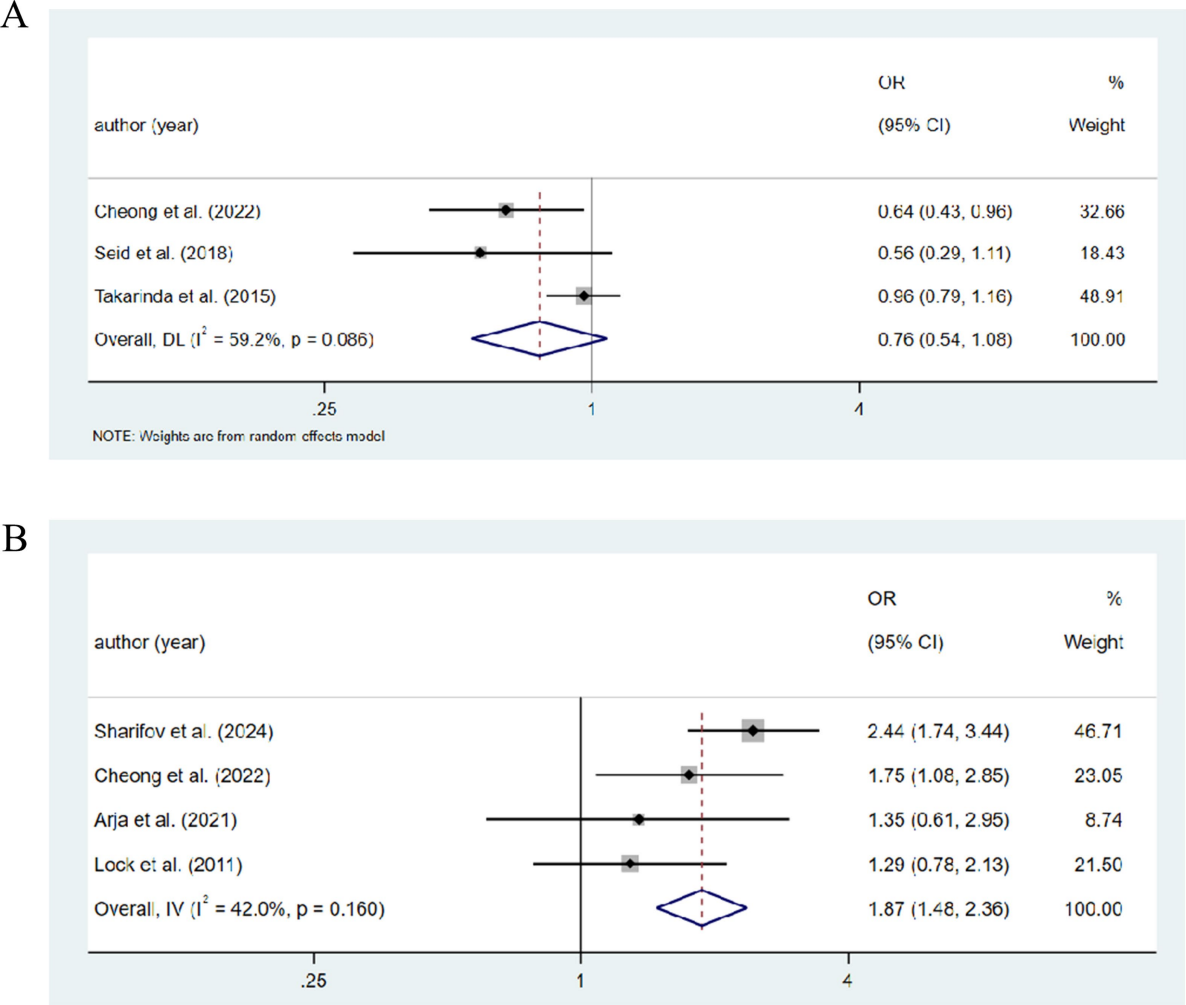
**(A)** Association of knowing someone with TB with patient delay; **(B)** Association of stigma with patient delay.

**Figure 10:**
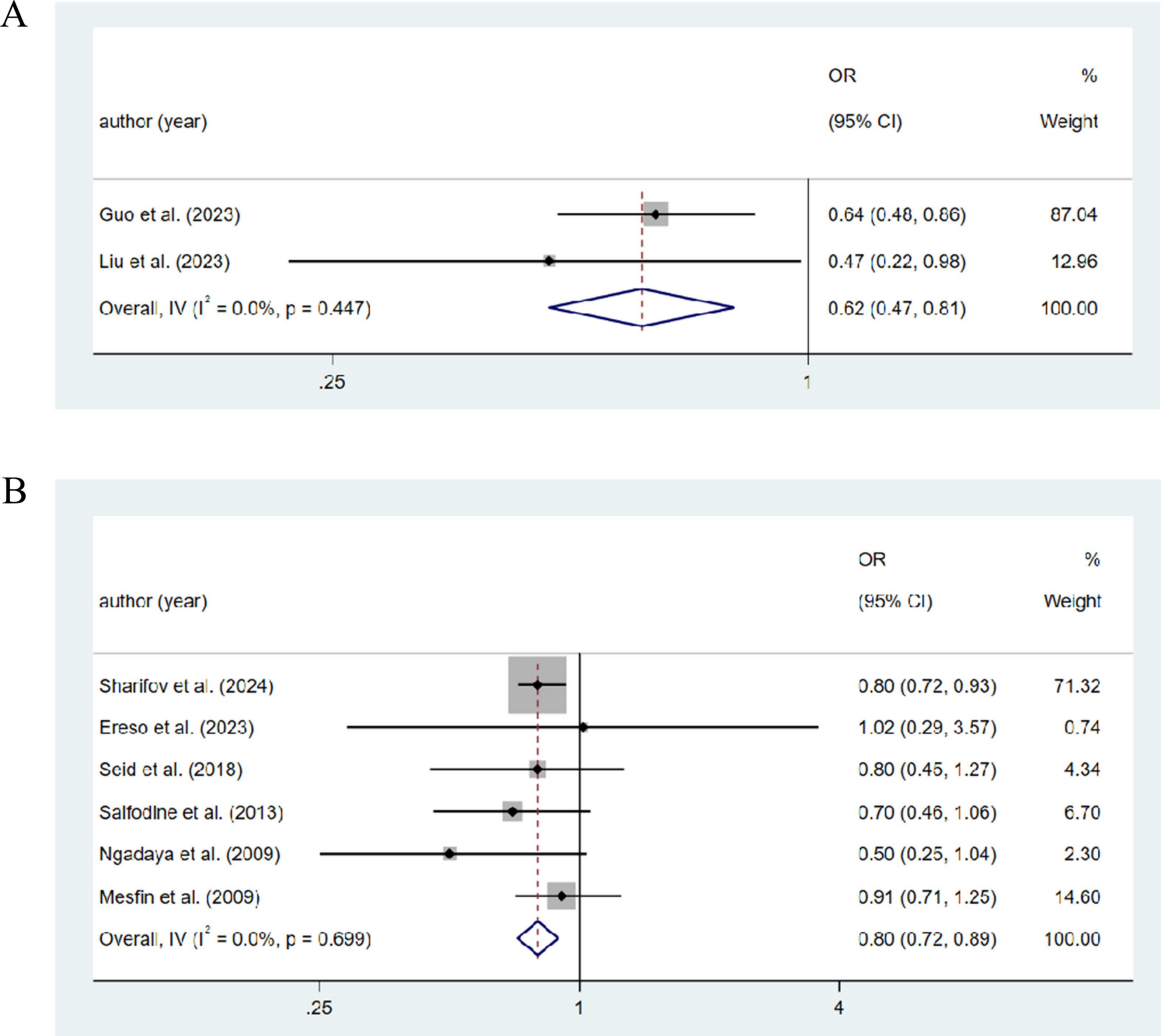
**(A)** Association of diabetes with patient delay; **(B)** Association of HIV status with patient delay.

#### 3.3.1 Personal factors

##### 3.3.1.1 Age (<25 Y vs. >25 Y)

Three studies (9, 39, 43) involving 1799 patients were included. We employed a fixed-effects model (*I*^2^ = 44.8%, *p* = 0.163), and found no association of age with patient delay (OR 0.96, 95% CI 0.84–1.10, *p* = 0.544), with a statistically insignificant difference.

##### 3.3.1.2 Sex (female vs. male)

Twenty-five studies (6–9, 22–25, 28–30, 33, 34, 38, 40, 42–44, 46–48, 54, 55, 57, 58)involving 145,851 patients were included. We employed a random-effects model (*I*^2^ = 87.0%, *p* = 0.000), and found no association of sex with patient delay (OR 1.03, 95% CI 0.96–1.11, *p* = 0.405), with a statistically insignificant difference.

##### 3.3.1.3 Occupation (farmer vs. non-farmer)

Eleven studies (5, 9, 25, 26, 29, 31, 34, 38, 39, 46, 55)involving 113,318 patients were included. We employed a random-effects model (*I*^2^ = 76.8%, *p* = 0.000), and found a close association of occupation with patient delay (OR 1.20, 95% CI 1.10–1.30, *p* < 0.001), with a statistically significant difference.

##### 3.3.1.4 Educational level (illiterate vs. literate)

Eleven studies (29, 34, 38, 39, 45, 46, 49, 53, 54, 57, 58) involving 7,079 patients were included. A random-effects model was adopted (*I*^2^ = 66.4%, p = 0.000). We found a close association of educational level with patient delay (OR 1.37, 95% CI 1.07–1.76, *p* = 0.012), with a statistically significant difference.

##### 3.3.1.5 Marital status (single vs. married)

Nine studies (6, 22, 38, 40, 46, 54–57)involving 2,917 patients were included. We employed a fixed-effects model (*I*^2^ = 6.2%, *p* = 0.383), and found no association of marital status with patient delay (OR 0.92, 95% CI 0.84–1.00, *p* = 0.054), with a statistically insignificant difference.

##### 3.3.1.6 BMI (BMI < 19 vs. 19 ≤ BMI ≤ 24)

Two studies (34, 39)involving 3,697 patients were included. We employed a fixed-effects model (*I*^2^ = 32.9%, *p* = 0.222), and found no association of BMI with patient delay (OR 1.26, 95% CI 0.89–1.80, *p* = 0.197), with a statistically insignificant difference.

#### 3.3.2 External factors

##### 3.3.2.1 Place of residence (rural vs. urban)

It was reported in 12 studies (22, 26, 29, 31, 39, 40, 43, 46, 52, 53, 55, 58)involving 5,070 patients. A fixed-effects model was adopted (*I*^2^ = 0.0%, *p* = 0.869). A close association of place of residence with patient delay was observed (OR 1.66, 95% CI 1.44–1.92, *p* < 0.001).

##### 3.3.2.2 Insurance (Yes vs. No)

It was reported in two studies (34, 50) involving 3,756 patients. We adopted a random-effects model (*I*^2^ = 58.2%, *p* = 0.122), and observed no association of insurance with patient delay (OR 1.45, 95% CI 0.83–2.55, *p* = 0.192).

##### 3.3.2.3 Smoking (smoker vs. non-smoker)

It was reported in six studies (9, 34, 38, 44, 46, 49) involving 6,141 patients. A random-effects model was adopted (*I*^2^ = 89.2%, *p* = 0.000). No association of smoking with patient delay was observed (OR 1.34, 95% CI 0.96–1.87, *p* = 0.081), with a statistically insignificant difference.

##### 3.3.2.4 Drinking (drinker vs. non-drinker)

It was reported in seven studies (9, 31, 34, 38, 44, 46, 49) involving 19,910 patients. A fixed-effects model was adopted (*I*^2^ = 46%, *p* = 0.099). We observed a close association of drinking with patient delay (OR 1.43, 95% CI 1.29–1.59, *p* < 0.001), with a statistically significant difference.

##### 3.3.2.5 Time of arrival at health services (>30 min vs. <30 min)

It was reported in four studies (29, 39, 43, 54) involving 764 patients. A random-effects model was adopted (*I*^2^ = 60.3%, *p* = 0.056). We found a close association of time of arrival at health services with patient delay (OR 1.34, 95% CI 1.03–1.74, *p* = 0.03).

##### 3.3.2.6 Distance to health services (>10 km vs. <10 km)

It was reported in four studies (29, 41, 45, 46) involving 1808 patients. A fixed-effects model was adopted (*I*^2^ = 5.9%, *p* = 0.363). We observed that distance to health services had no association with patient delay (OR 1.13, 95% CI 0.91–1.39, *p* = 0.266), with a statistically insignificant difference.

#### 3.3.3 Disease-related factors

##### 3.3.3.1 TB classification (smear-negative vs. smear-positive)

It involved seven studies (9, 24, 25, 27, 30, 39, 43) with 129,829 patients. We employed a random-effects model (*I*^2^ = 94.2%, *p* = 0.000), and observed a close association of TB classification with patient delay (OR 1.31, 95% CI 1.15–1.49, *p* < 0.001).

##### 3.3.3.2 TB knowledge (No vs. Yes)

It involved six studies (9, 29, 31, 40, 47, 49) with 3,186 patients. We employed a random-effects model (*I*^2^ = 61.6%, *p* = 0.023), and observed a significant association of TB knowledge with patient delay (OR 2.09, 95% CI 1.61–3.71, *p* < 0.001).

##### 3.3.3.3 Knowing someone with TB (Yes vs. No)

It involved three studies (37, 43, 44) with 1,497 patients. We employed a random-effects model (*I*^2^ = 59.2%, *p* = 0.086), and observed no association of knowing someone with TB with patient delay (OR 0.76, 95% CI 0.54–1.08, *p* = 0.127), with a statistically insignificant difference.

##### 3.3.3.4 Stigma (Yes vs. No)

It involved four studies (28, 37, 39, 42)with 1,571 patients. We utilized a fixed-effects model (*I*^2^ = 42.0%, *p* = 0.160), and observed a close association of stigma with patient delay (OR 1.87, 95% CI 1.48–2.36, *p* < 0.001), with a statistically significant difference.

##### 3.3.3.5 Diabetes (no diabetes vs. diabetes)

It involved two studies (5, 34) with 6,632 patients. We utilized a fixed-effects model (*I*^2^ = 0.0%, *p* = 0.447), and observed a close association of diabetes with patient delay (OR 0.62, 95% CI 0.47–0.81, *p* < 0.001), showing a statistically significant difference.

##### 3.3.3.6 HIV status (HIV-negative vs. HIV-positive)

It involved six studies (9, 28, 43, 50, 54, 55) with 3,705 patients. We utilized a fixed-effects model (*I*^2^ = 0.0%, *p* = 0.699), and observed a close association of HIV status with patient delay (OR 0.80, 95% CI 0.72–0.89, *p* = 0.032), showing a statistically significant difference.

##### 3.3.3.7 Sensitivity analyses

The leave-one-out sensitivity analyses revealed no significant changes in the direction and significance of the pooled effect size. Therefore, the pooled results of this meta-analysis were relatively robust (Appendix 2).

##### 3.3.3.8 Publication bias

**Figure 11:**
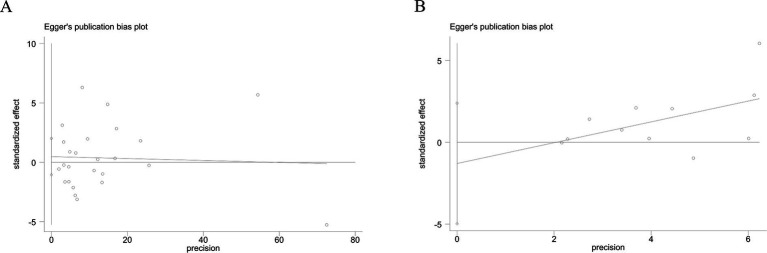
**(A)** Publication bias for sex; **(B)** Publication bias for educational level.

**Figure 12:**
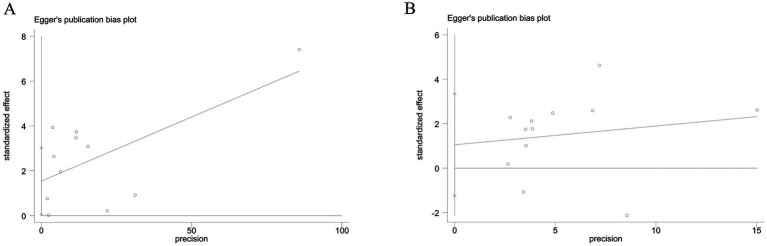
**(A)** Publication bias for occupation; **(B)** Publication bias for place of residence.

**Figure 13:**
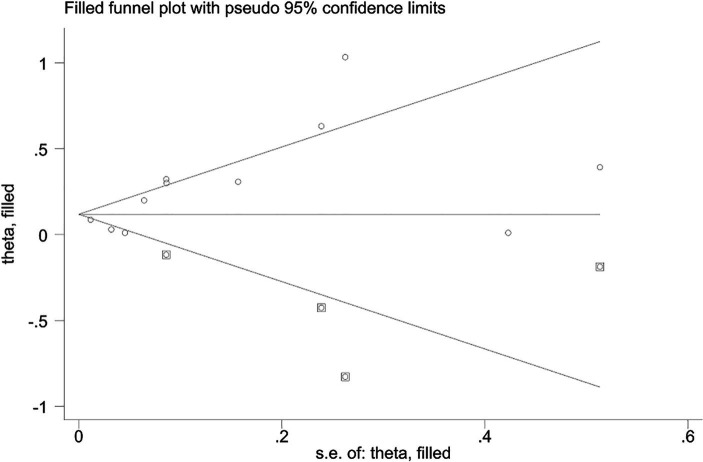
Publication bias for place of residence.

Only sex, educational level, occupation, and place of residence were assessed for publication bias (Figures 11, 12). The results showed no potential publication bias for sex (*p* = 0.527), educational level (*p* = 0.447), and place of residence (*p* = 0.96), but occupation had a potential publication bias (*p* = 0.043). The other factors were not assessed because no more than ten studies were included. Using the trim-and-fill method, we observed no significant change in the results for place of residence (p = 0.043), suggesting that the results were relatively robust (Figure 13).

## 4 Discussion

This is the first meta-analysis globally synthesizing multiple determinants of TB patient delay across diverse settings. 42 studies (two case–control, seven cohort, and 33 cross-sectional studies) were included. Occupation, educational level, place of residence, TB classification, diabetes, HIV infection, drinking, TB knowledge, stigma, and time of arrival at health services were all significantly correlated with patient delay.

This study revealed a close association of patient delay with occupation. This may be related to farmers’ long working time, limited access to healthcare, and a lack of knowledge regarding TB prevention and treatment, consistent with the findings by Obeagu (59). At the patient level, patient delay is associated with educational level. In this paper, patient delay in the illiterate group was 1.25 times that in the literate group, consistent with the meta-analysis results by Getahun Fetensa (60) in Ethiopia. This suggests that educational level is associated with patients’ ability to quickly recognize their symptoms, the degree of their health awareness, and medical treatment status. However, the heterogeneity was up to 81.4%, possibly related to the information derived from different countries. Moreover, rural residents were 1.66 times more prone to TB delay than urban residents, consistent with the findings by Li Y, which is related to traffic inconvenience, low awareness of healthcare, and lack of medical resources in rural areas (61).

Sputum smear is a commonly used diagnostic method in hospitals, but it cannot be used to definitively diagnose TB. The reason is that TB has an incubation period ranging from 4 to 8 weeks to several decades, and external factors can also influence the specimens submitted for testing. In this paper, the results revealed that patient delay occurred in sputum smear-negative patients 1.66 times more than in smear-positive patients. The misdiagnosis and missed diagnosis of sputum smear-negative TB cases were associated with the accuracy of pathogen-based tests such as sputum smear microscopy and bacterial culture. This is consistent with the findings reported by N. Lorent (62) that patient delay is related to the classification of TB. It was also found that diabetic patients had a 38% lower patient delay rate than non-diabetic patients, in line with the findings by Anees ur Rehman (63). The possible reason is that diabetic patients usually receive follow-up from a same local physician every quarter, improving early detection. In terms of HIV status, the risk of delay in HIV-negative patients was 20% of that in HIV-positive ones, similar to the results by Rachael M Burke (64). Since all patients receiving HIV-related services are actively provided with TB information, HIV-infected patients are more likely to be educated about TB. Actually, more HIV-positive patients possessed adequate TB knowledge than HIV-negative ones in this study. Additionally, patient delay was 1.43 times more likely to occur in drinkers than in non-drinkers, consistent with evidence worldwide that drinking is associated with global animal mortality (65).

Early, regular, complete, appropriate, and combined treatment of TB is important, so early detection is necessary, which requires a better understanding of TB. In this paper, patients with TB knowledge had 2.25 times lower risk of patient delay than those without TB knowledge, consistent with the results by Marie Varughese (66). This highlights the need to strengthen health education to improve public understanding. As TB is an infectious disease, normal people tend to avoid contact with these patients. TB patients often worry about social discrimination and are estranged from their families, so stigma is associated with patient delay. As a result, stigma has an influence on TB patients to a certain extent. In this paper, patients with high stigma had 1.48 times higher risk of delay than those with low stigma, in line with the findings by Hailay Abrha Gesesew (67). Additionally, the time of arrival at health services influenced patients’ health-seeking behavior to a certain extent, consistent with the meta-analysis by Marie Varughese (66) that time of arrival at health services is a risk factor of patient delay. The reason is that transportation costs incurred by long journeys impose a direct financial burden on poor patients in underdeveloped regions. In developed regions with a rapid pace of life, the time cost of round-trip travel is extremely high for residents who need to work and care for their families. A meta-study (68) revealed significant associations of sex and marital status with patient delay, but the two factors showed no influence in this paper, possibly related to the different data across countries. In developed countries and some developing countries with good economic conditions, men and women are granted equal status, hold open attitudes toward marriage, and obtain equal access to social resources. In contrast, married women in poor and backward countries focus more on their families than men and spend no time on their health, leading to patient delay. Single women gain less social support than married women, resulting in patient delay. A meta-analysis by Marie Varughese (66) showed that distance to health services is a risk factor for patient delay. However, this association was not observed in this study, probably because of less rigorous stratification of relative distances and the limited number of included studies.

In summary, despite advances in healthcare, TB remains a major global public health issue, and patient delay is one of the key causes of the TB spread. Clinically, pulmonary lesions will progressively aggravate following TB patient delay, resulting in cavity and fibrosis, and causing irreversible damage to lung function. In severe cases, the disease can spread systemically to lead to complications such as tuberculous meningitis and bone TB, increasing the risk of death. Long-term failure to adhere to standardized medication can also produce drug resistance in *Mycobacterium tuberculosis*, rendering standard anti-TB drugs ineffective. As a result, the treatment duration is prolonged, and the cure rate significantly declines. Socially, patients suffering from long-term low-grade fever, cough, and weakness are unable to work or live normally, with reduced labor capacity. The high costs of long-term treatment also place a heavy financial burden on families, often resulting in poverty caused by illness. Additionally, the resulting physical discomfort and social isolation can lead to negative psychological states. From a public health perspective, TB excreters with patient delay remain sources of infection and continuously spread the bacteria to the people around, infecting their families and colleagues, and increasing the risk of cluster outbreaks. Moreover, drug-resistant *Mycobacterium tuberculosis* also spreads among people, leading to the spread of refractory TB. This will increase the workload in community screening, follow-up, and management, and complicate the overall prevention and control of TB. Therefore, identifying risk factors for TB patient delay is crucial for minimizing delay and achieving early detection and effective treatment, which is key to successful TB control.

However, the following limitations are worth noting. First, this is a meta-analysis of prevalence surveys, and most of the included studies were cross-sectional in design, so the subjects were susceptible to subjective factors, limiting causal inferences. Second, no more than 10 studies were included for many factors, so they were not assessed for publication bias. Third, the included studies encompassed data from different countries with various economic levels, lifestyles, and customs, which may influence the study results for patient delay. In the future, more in-depth analyses are required based on different regions. Fourth, only English-language studies were included, potentially introducing language bias. Fifth, patient delay had different definitions across studies, leading to high heterogeneity.

## 5 Conclusion

This meta-analysis demonstrates that the TB patient delay rate remains high across countries. Occupation, educational level, place of residence, TB classification, diabetes, HIV infection, drinking, TB knowledge, and stigma are all significantly associated with patient delay. In future prevention and control, it is necessary to make early interventions in at-risk populations, build a perfect health service system in rural areas, enhance TB health education, raise awareness of the disease, and implement early screening.

## Data Availability

The original contributions presented in the study are included in the article/Supplementary material, further inquiries can be directed to the corresponding author.
