# Supplementary material for: Factors associated with tuberculosis patient delay: a meta-analysis
Source: Front Public Health. 2026 Jul 14;14:1832410. doi: 10.3389/fpubh.2026.1832410 (PMC13407767; doi:10.3389/fpubh.2026.1832410)
Supplement: Supplementary file 1 [file Data_Sheet_1.DOCX]

# Appendix 1: Search strategy for network meta-analysis.

## Search strategy of Pubmed.

| No. | Search items |
| --- | --- |
| #1 | "Tuberculosis, Pulmonary"[Mesh] |
| #2 | bronchial tuberculos* [Title/Abstract] OR bronchus tuberculosis [Title/Abstract] OR cavernous tuberculosis [Title/Abstract] OR cavitary tuberculosis [Title/Abstract] OR chronic lung tuberculosis [Title/Abstract] OR chronic pulmonary tuberculosis [Title/Abstract] OR coniotuberculosis [Title/Abstract] OR lung caseation [Title/Abstract] OR lung TB[Title/Abstract] OR lung tuberculosis [Title/Abstract] OR phthisis[Title/Abstract] OR pneumonophthisis[Title/Abstract] OR pneumophthisiology[Title/Abstract] OR Pulmonary Consumption*[Title/Abstract] OR Pulmonary Phthis*[Title/Abstract] OR pulmonary TB[Title/Abstract] OR Pulmonary Tuberculos*[Title/Abstract] OR tiocarlide chronic lung tuberculosis[Title/Abstract] OR tuberculosis bronchi*[Title/Abstract] OR tuberculosis pulmonis[Title/Abstract] OR lung tuberculous cavity[Title/Abstract] OR lung tuberculosis treatment[Title/Abstract] |
| #3 | #1 OR #2 |
| #4 | delay*[Title/Abstract] OR diagnosis Delay* [Title/Abstract] OR delayed consultation[Title/Abstract] OR patient delay*[Title/Abstract] OR healthcare seeking[Title/Abstract] OR provider delay* [Title/Abstract] OR doctor delay*[Title/Abstract] OR health system delay*[Title/Abstract] OR late diagnos*[Title/Abstract] |
| #5 | #3 AND #4 |

## Search strategy of Embase.

| No. | Search items |
| --- | --- |
| #1 | 'lung tuberculosis'/exp |
| #2 | 'bronchial tuberculos*':ab,ti OR 'bronchus tuberculosis':ab,ti OR 'cavernous tuberculosis':ab,ti OR 'cavitary tuberculosis':ab,ti OR 'chronic lung tuberculosis':ab,ti OR 'chronic pulmonary tuberculosis':ab,ti OR coniotuberculosis:ab,ti OR 'lung caseation':ab,ti OR 'lung tb':ab,ti OR 'lung tuberculosis':ab,ti OR phthisis:ab,ti OR pneumonophthisis:ab,ti OR pneumophthisiology:ab,ti OR 'pulmonary consumption*':ab,ti OR 'pulmonary phthis*':ab,ti OR 'pulmonary tb':ab,ti OR 'pulmonary tuberculos*':ab,ti OR 'tiocarlide chronic lung tuberculosis':ab,ti OR 'tuberculosis bronchi*':ab,ti OR 'tuberculosis pulmonis':ab,ti OR 'lung tuberculous cavity':ab,ti OR 'lung tuberculosis treatment':ab,ti |
| #3 | #1 OR #2 |
| #4 | delay*:ab,ti OR 'diagnosis delay*':ab,ti OR 'delayed consultation':ab,ti OR 'patient delay*':ab,ti OR (healthcare AND seeking) OR 'provider delay*':ab,ti OR 'doctor delay*':ab,ti OR 'health system delay*':ab,ti OR 'late diagnos*':ab,ti |
| #5 | #3 AND #4 |

## Search strategy of Cochrane Library.

| No. | Search items |
| --- | --- |
| #1 | MeSH descriptor: [Tuberculosis, Pulmonary] explode all trees |
| #2 | (bronchial tuberculos* or bronchus tuberculosis or cavernous tuberculosis or cavitary tuberculosis or chronic lung tuberculosis or chronic pulmonary tuberculosis or coniotuberculosis or lung caseation or lung TB or lung tuberculosis or phthisis or pneumonophthisis or pneumophthisiology or Pulmonary Consumption* or Pulmonary Phthis* or pulmonary TB or Pulmonary Tuberculos* or tiocarlide chronic lung tuberculosis or tuberculosis bronchi* or tuberculosis pulmonis or lung tuberculous cavity or lung tuberculosis treatment):ti,ab,kw |
| #3 | #1 OR #2 |
| #4 | (delay* or diagnosis Delay* or delayed consultation or patient delay* or healthcare seeking or provider delay* or doctor delay* or health system delay* or late diagnos*):ti,ab,kw |
| #5 | #3 and #4 |

## Search strategy of Web of Science.

| No. | Search items |
| --- | --- |
| #1 | (bronchial tuberculos* (Topic) OR bronchus tuberculosis (Topic) OR cavernous tuberculosis (Topic) OR cavitary tuberculosis (Topic) OR chronic lung tuberculosis (Topic) OR chronic pulmonary tuberculosis (Topic) OR coniotuberculosis (Topic) OR lung caseation (Topic) OR lung TB (Topic) OR lung tuberculosis (Topic) OR phthisis (Topic) OR pneumonophthisis (Topic) OR pneumophthisiology (Topic) OR Pulmonary Consumption* (Topic) OR Pulmonary Phthis* (Topic) OR pulmonary TB (Topic) OR Pulmonary Tuberculos* (Topic) OR tiocarlide chronic lung tuberculosis (Topic) OR tuberculosis bronchi* (Topic) OR tuberculosis pulmonis (Topic) OR lung tuberculous cavity (Topic) OR lung tuberculosis treatment (Topic)) |
| #2 | (delay* (Topic) OR diagnosis Delay* (Topic) OR delayed consultation (Topic) OR patient delay* (Topic) OR healthcare seeking (Topic) OR provider delay* (Topic) OR doctor delay* (Topic) OR health system delay* (Topic) OR late diagnos* (Topic)) |
| #3 | #1 AND #2 |
