# Supplementary material for: Factors associated with tuberculosis patient delay: a meta-analysis
Source: Front Public Health. 2026 Jul 14;14:1832410. doi: 10.3389/fpubh.2026.1832410 (PMC13407767; doi:10.3389/fpubh.2026.1832410)
Supplement: Supplementary file 2 [file Data_Sheet_2.DOC]

**Appendix 2 Sensitivity analysis**


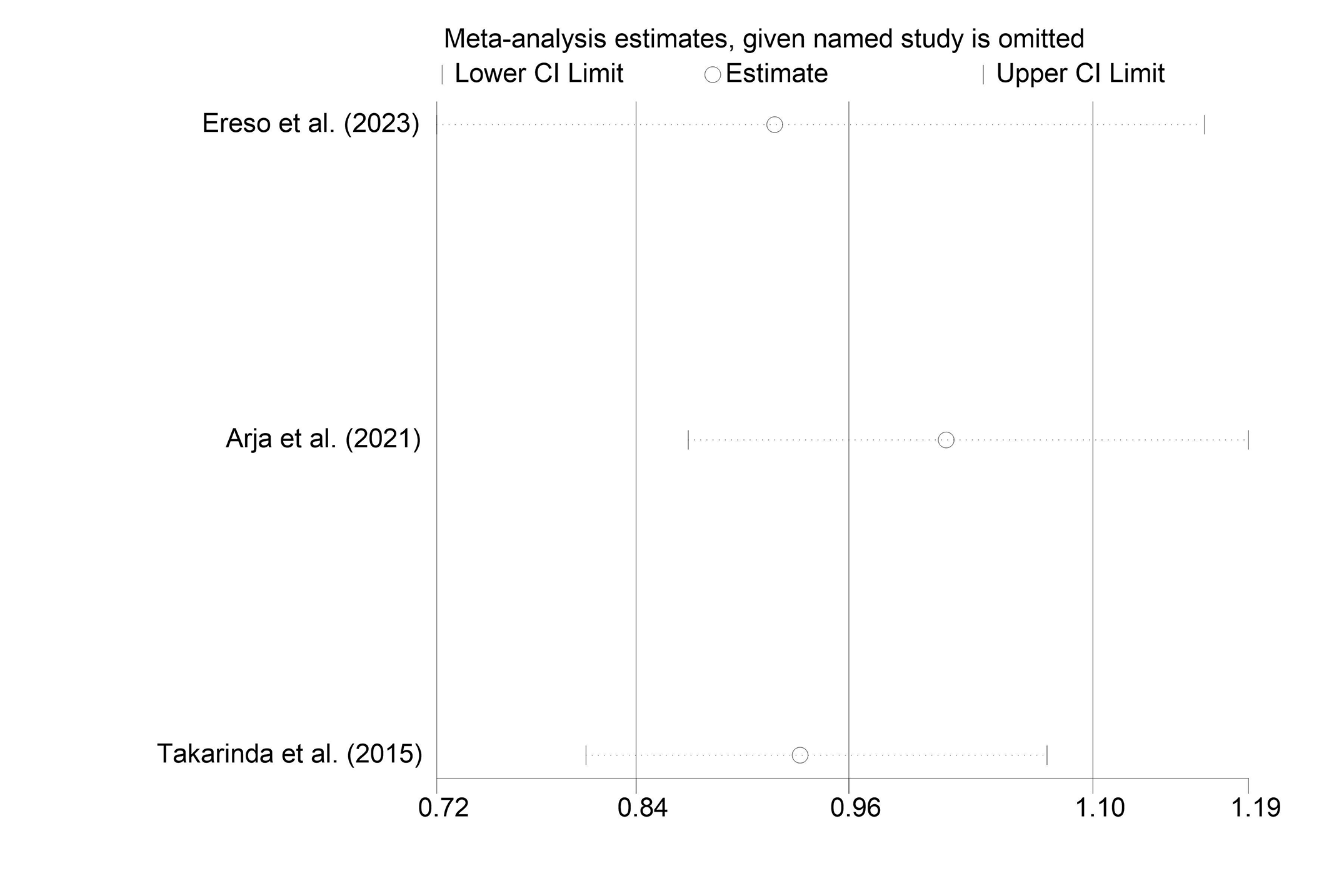


FigureS1. Sensitivity analysis for age,2025.

FigureS2. Sensitivity analysis for sex,2025.


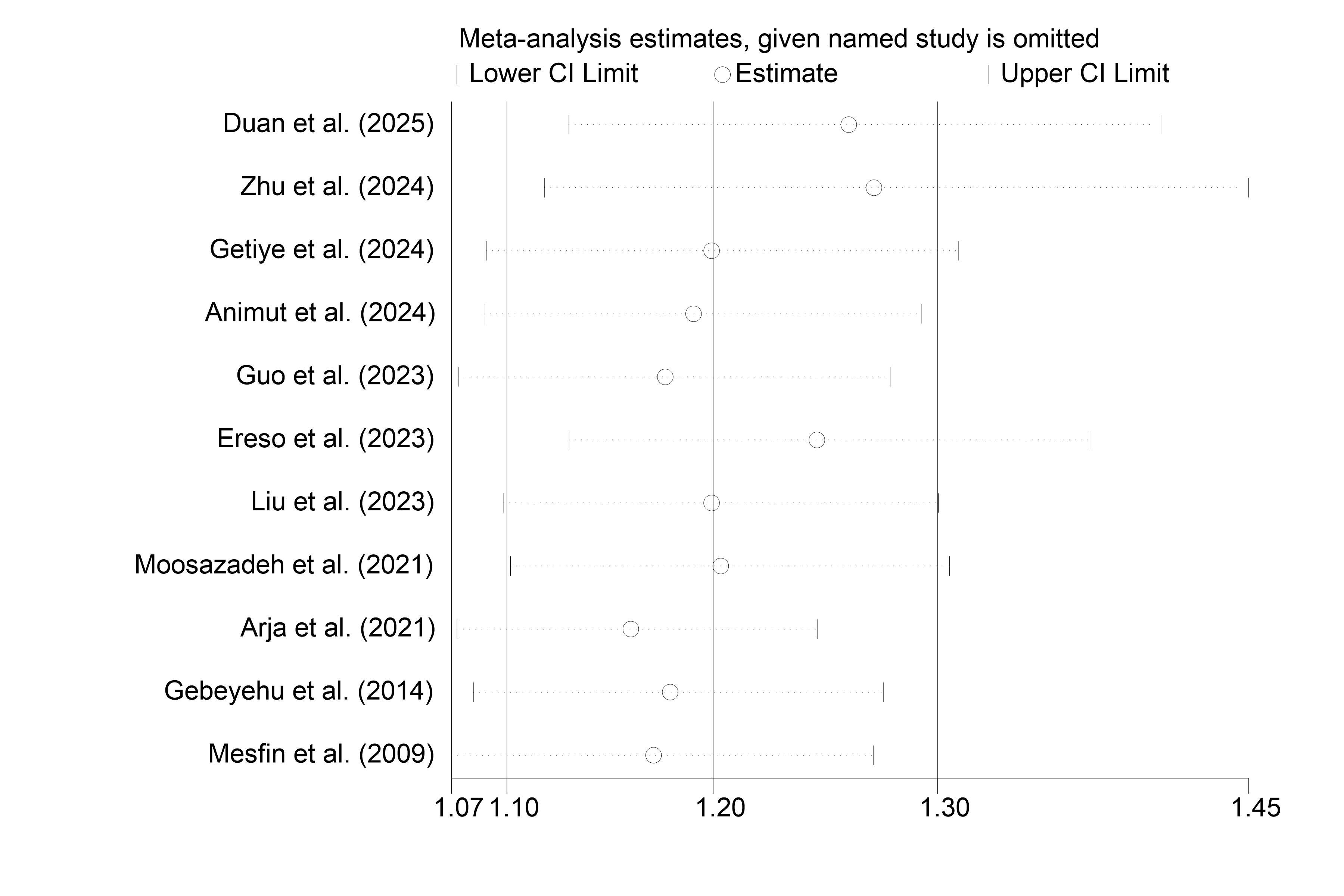


FigureS3. Sensitivity analysis for occupation,2025.


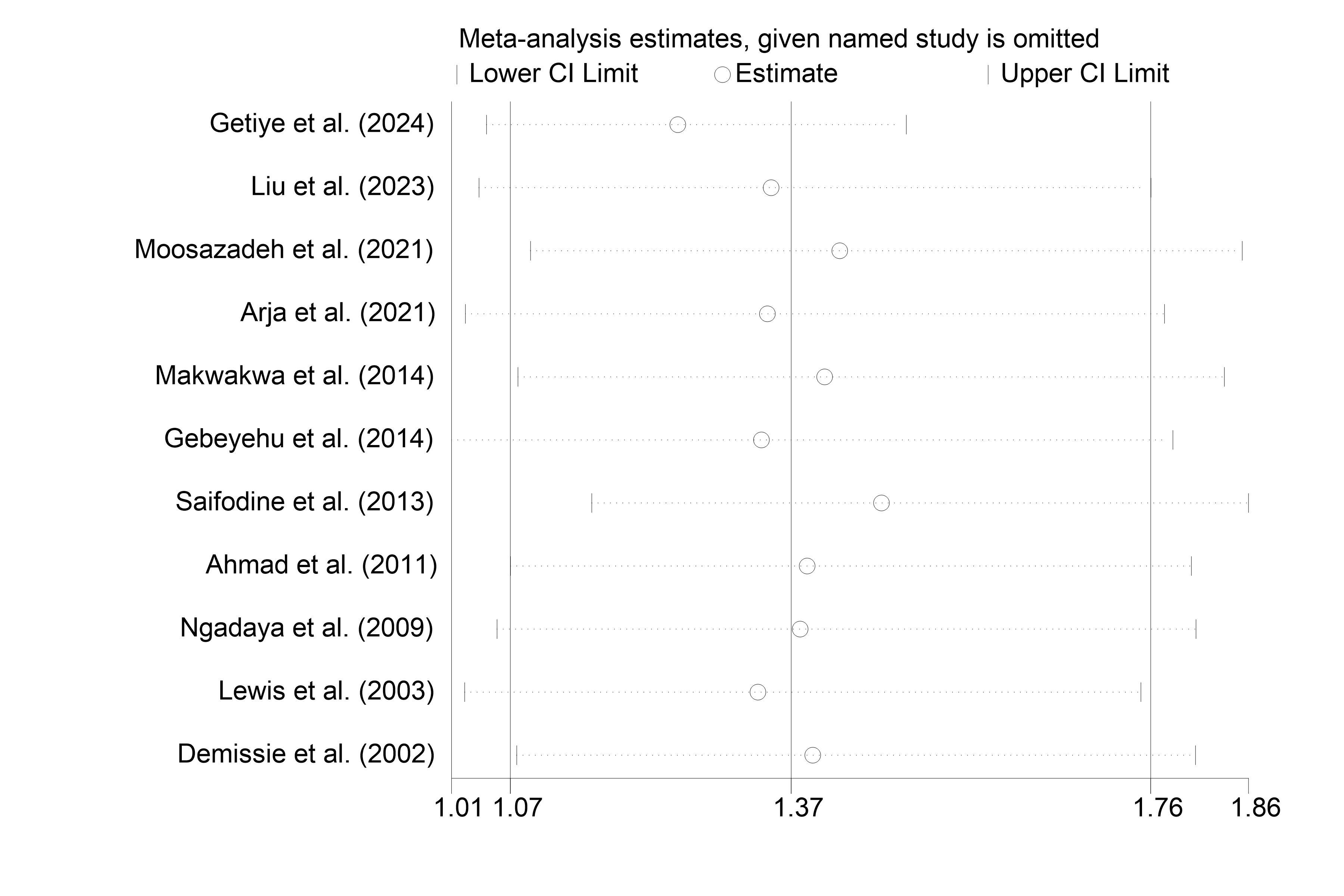


FigureS4. Sensitivity analysis for educational level,2025.


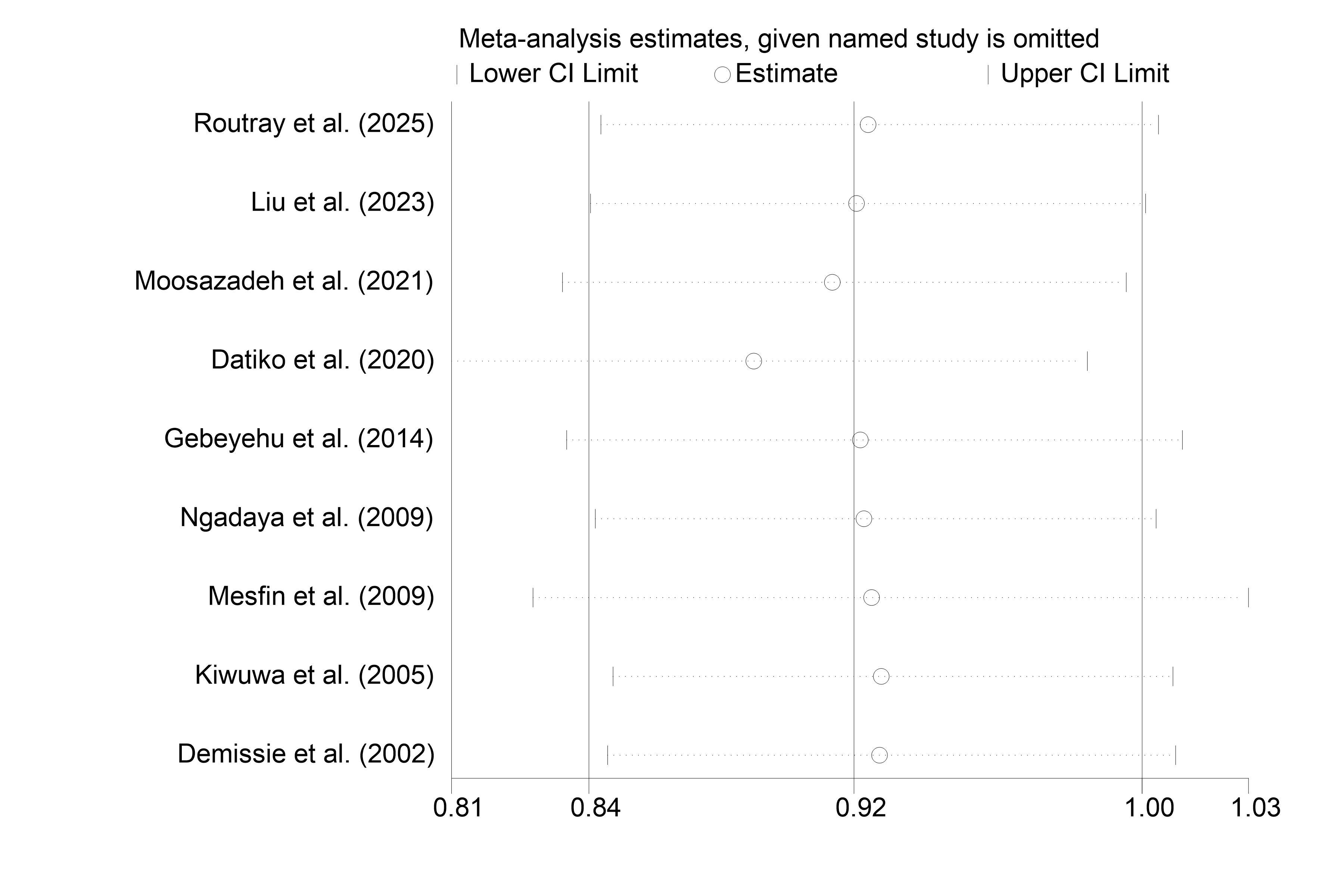


FigureS5. Sensitivity analysis for marital status,2025.


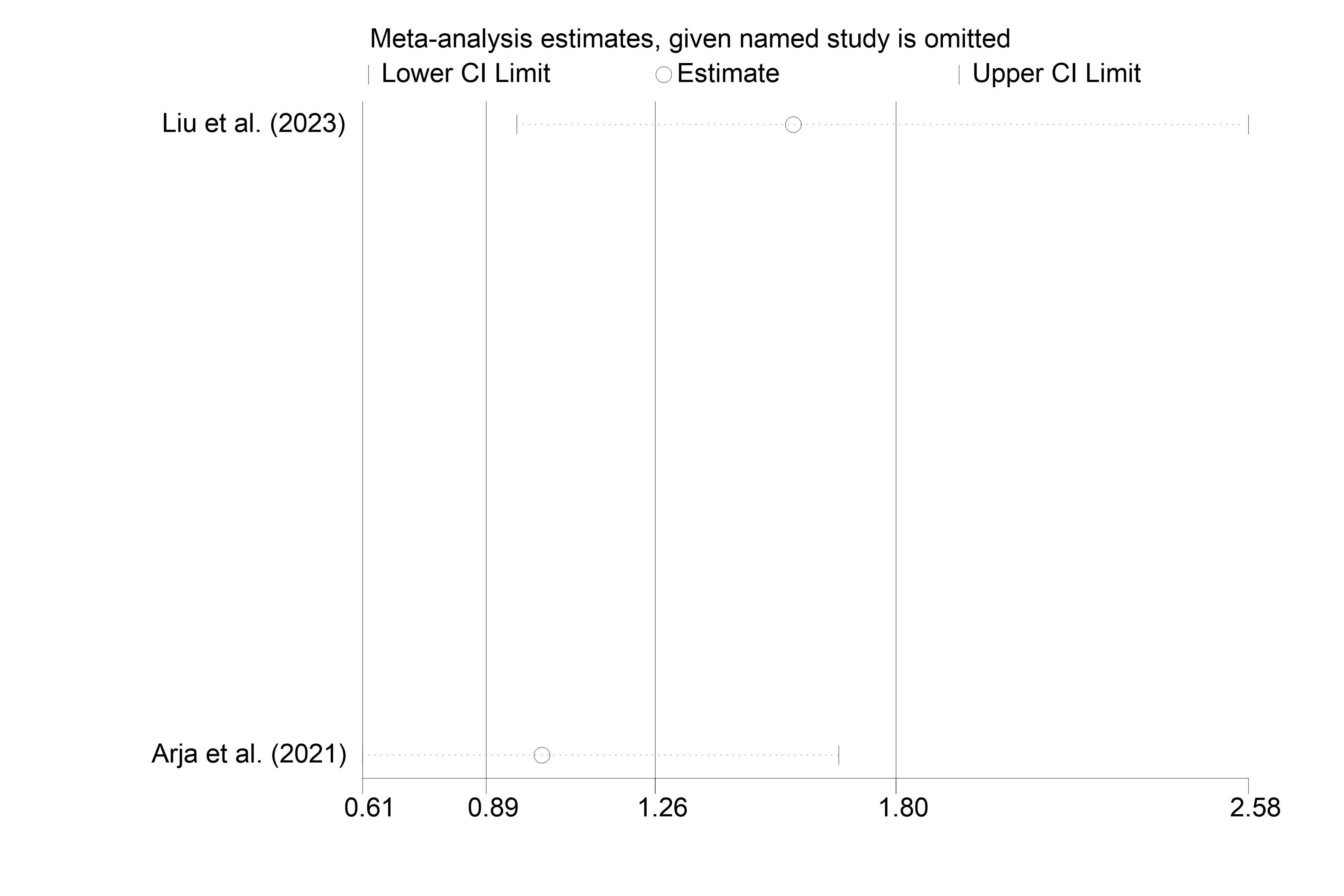


FigureS6. Sensitivity analysis for BMI,2025.


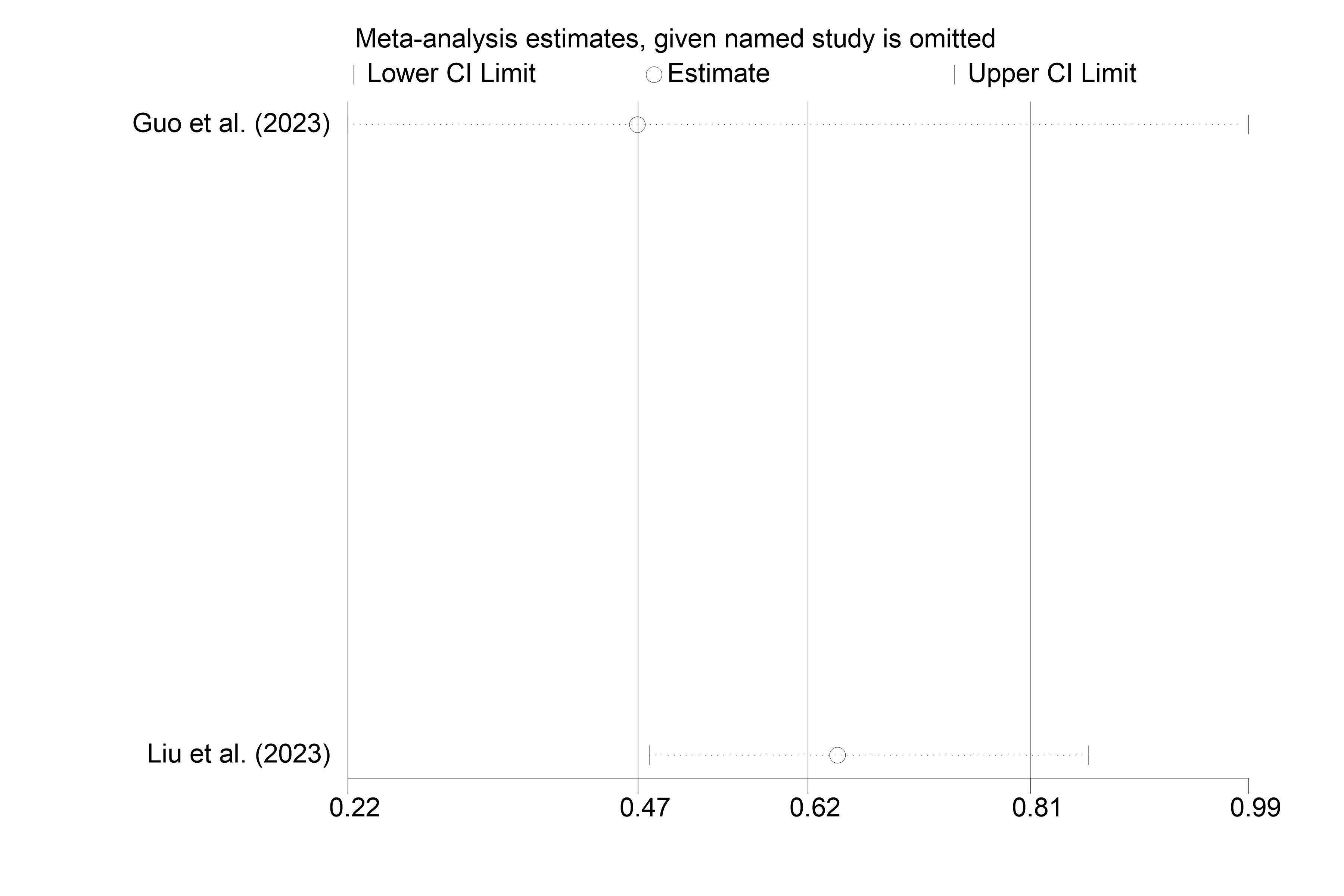


FigureS7. Sensitivity analysis for diabetes,2025.


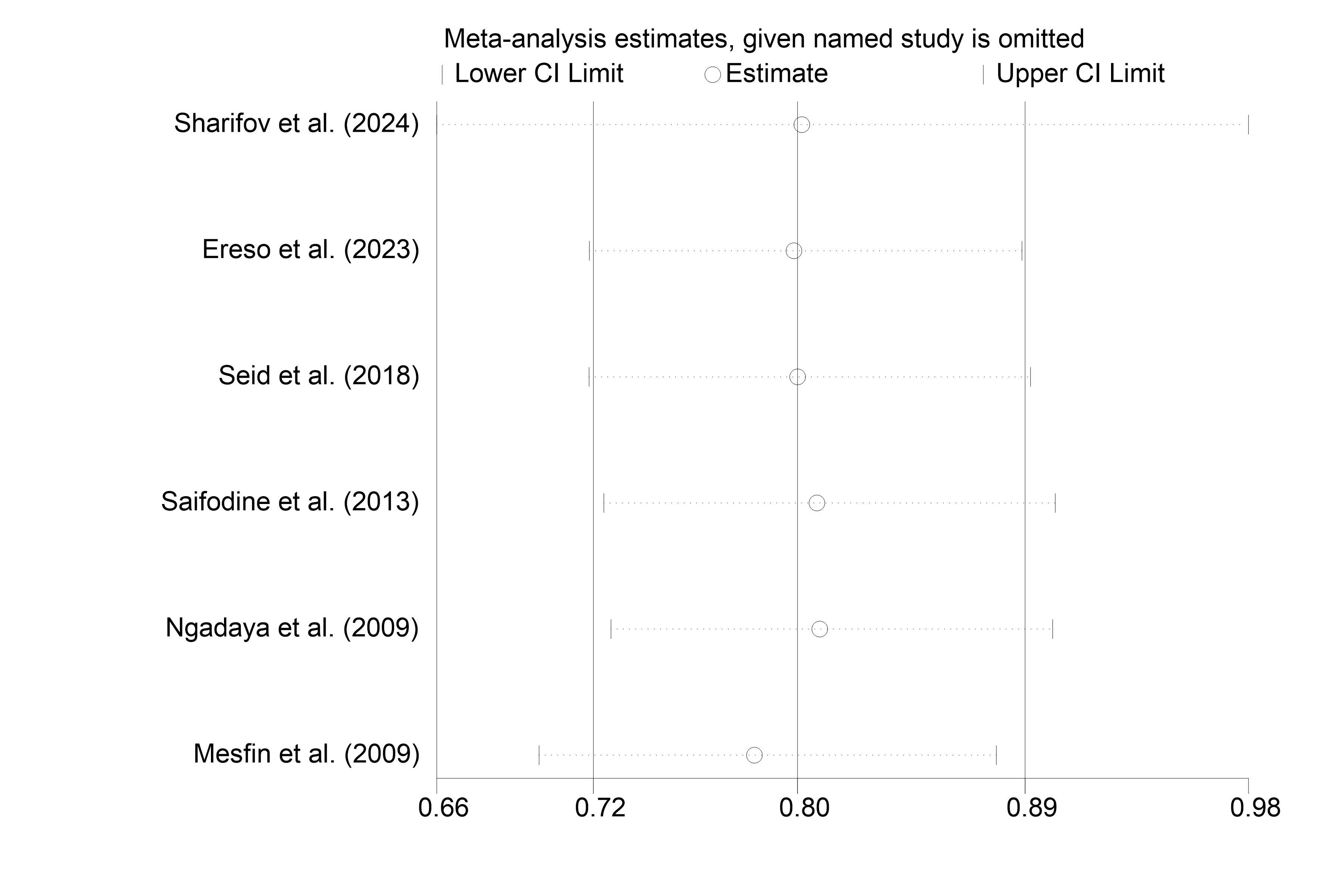


FigureS8. Sensitivity analysis for HIV status,2025.


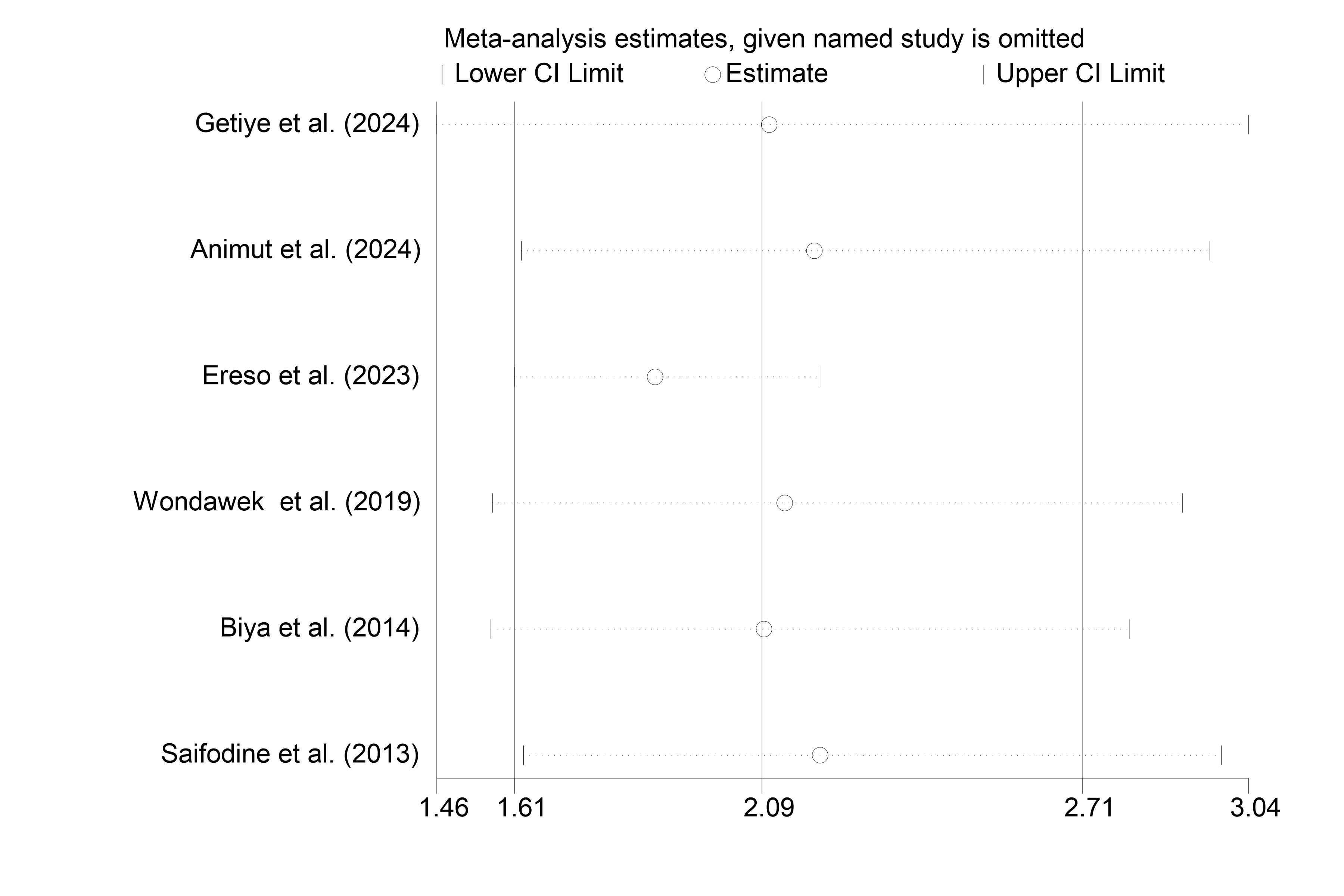


FigureS9. Sensitivity analysis for TB knowledge,2025.


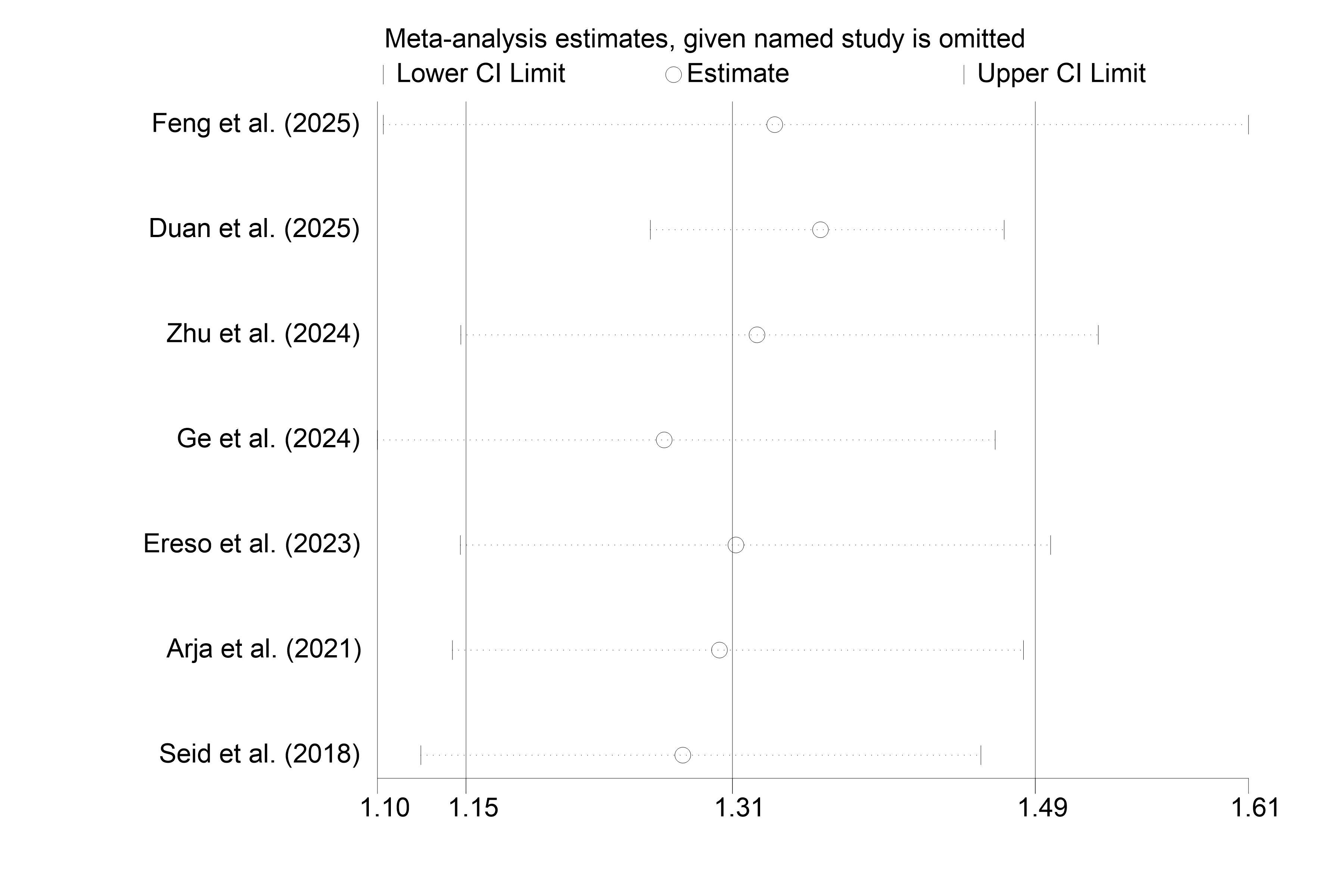


FigureS10. Sensitivity analysis for TB classification,2025.


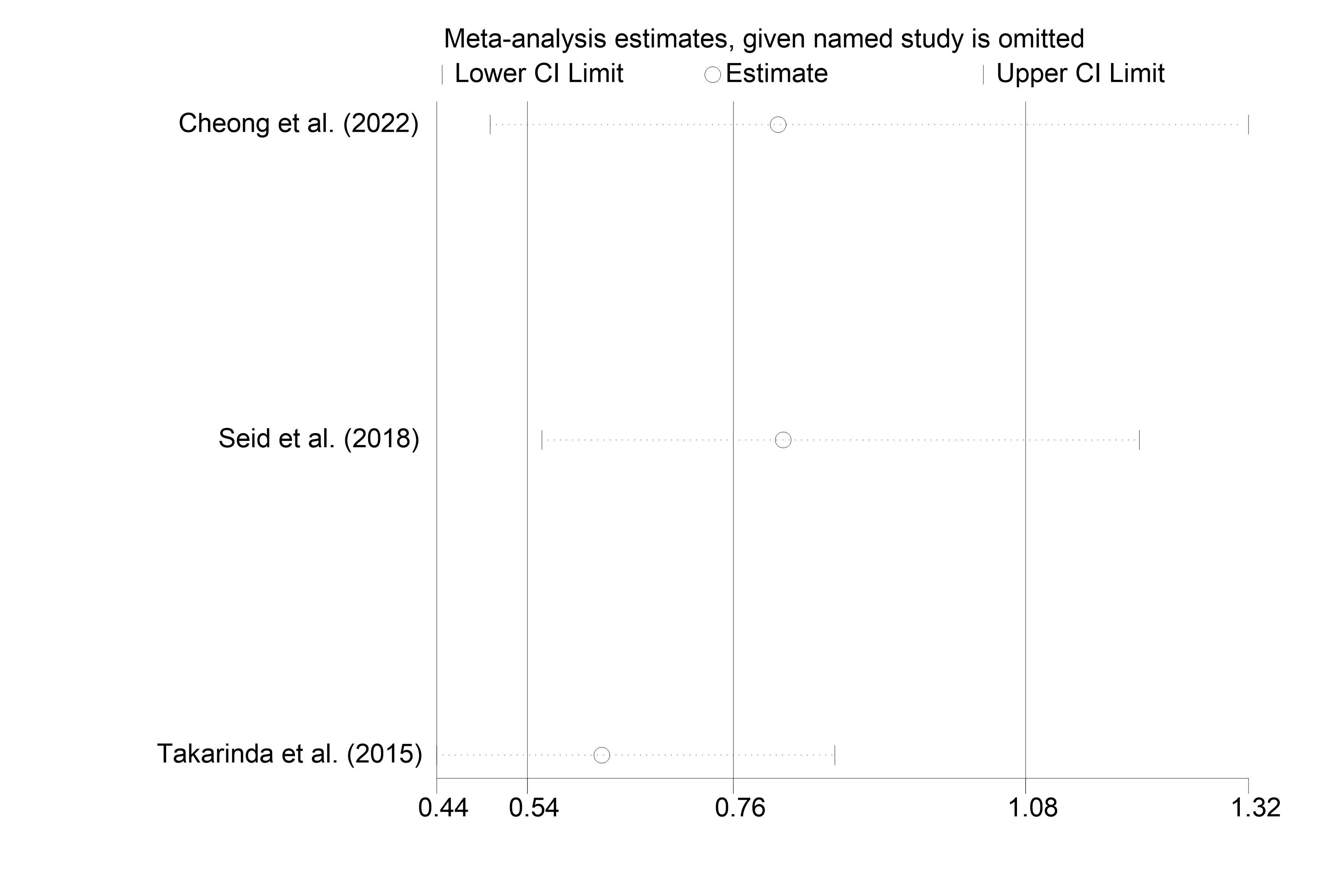


FigureS11. Sensitivity analysis for knowing someone with TB,2025.


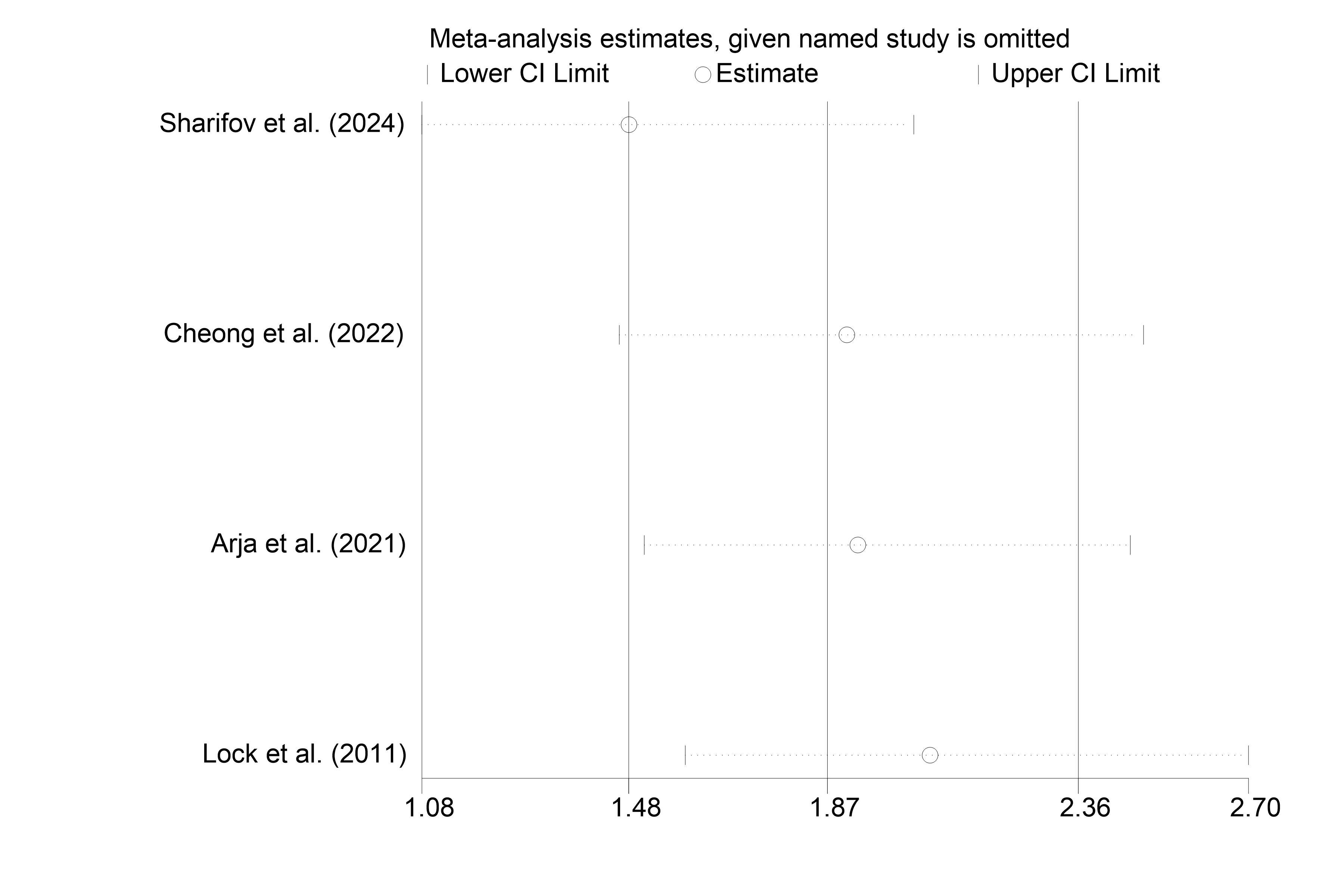


FigureS12. Sensitivity analysis for stigma,2025.


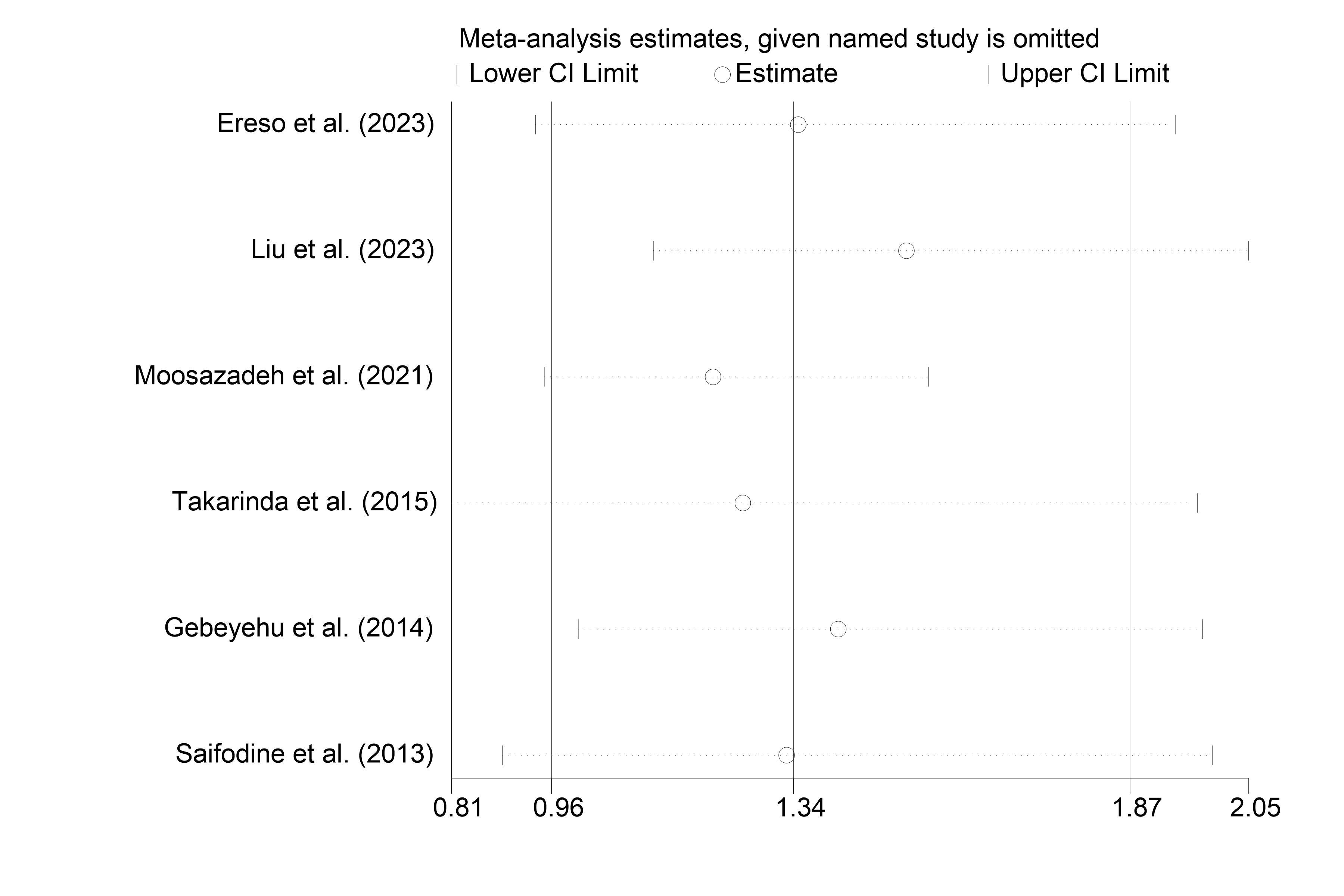


FigureS13. Sensitivity analysis for smoking,2025.


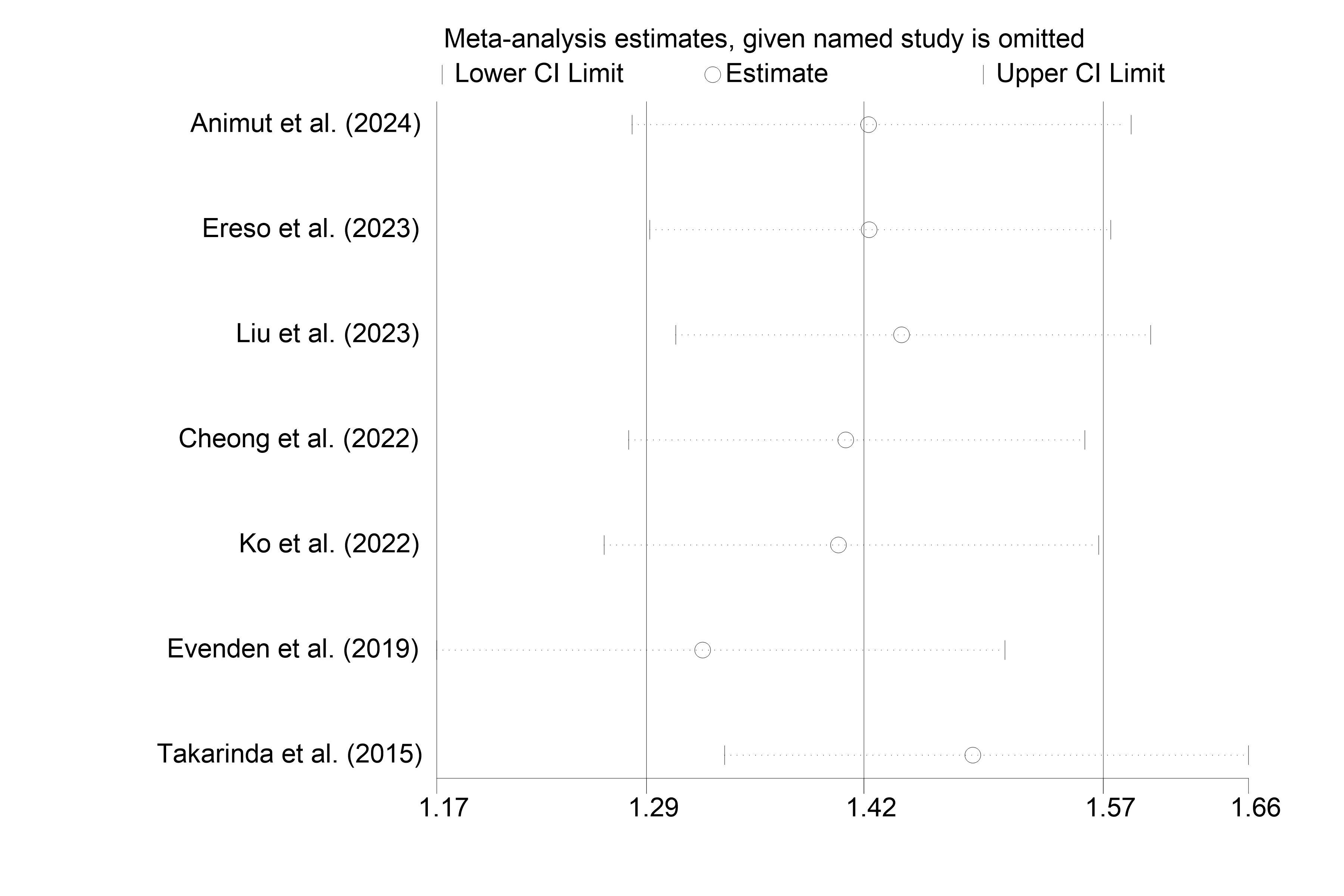


FigureS14. Sensitivity analysis for drinking,2025.


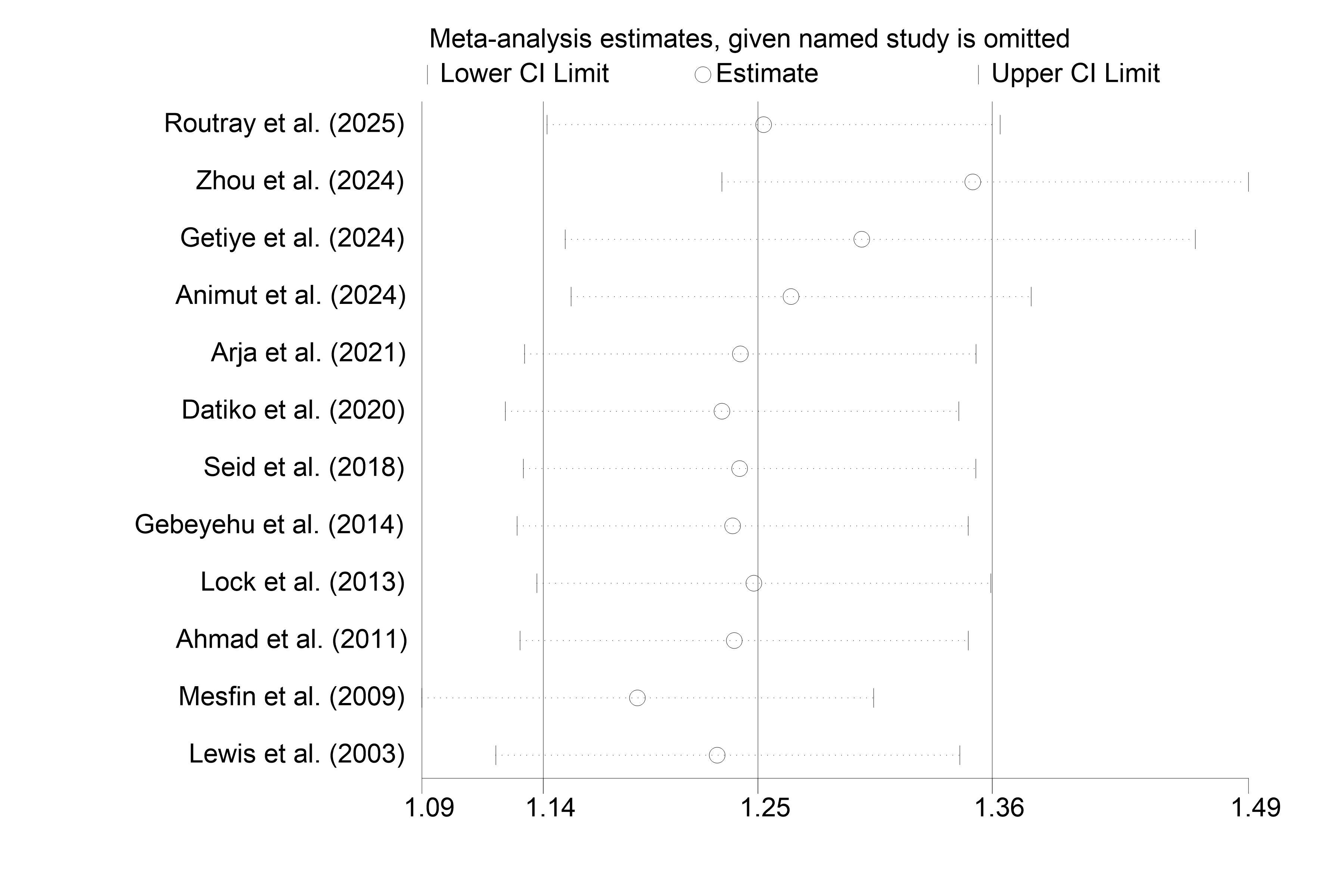


FigureS15. Sensitivity analysis for place of residence,2025.


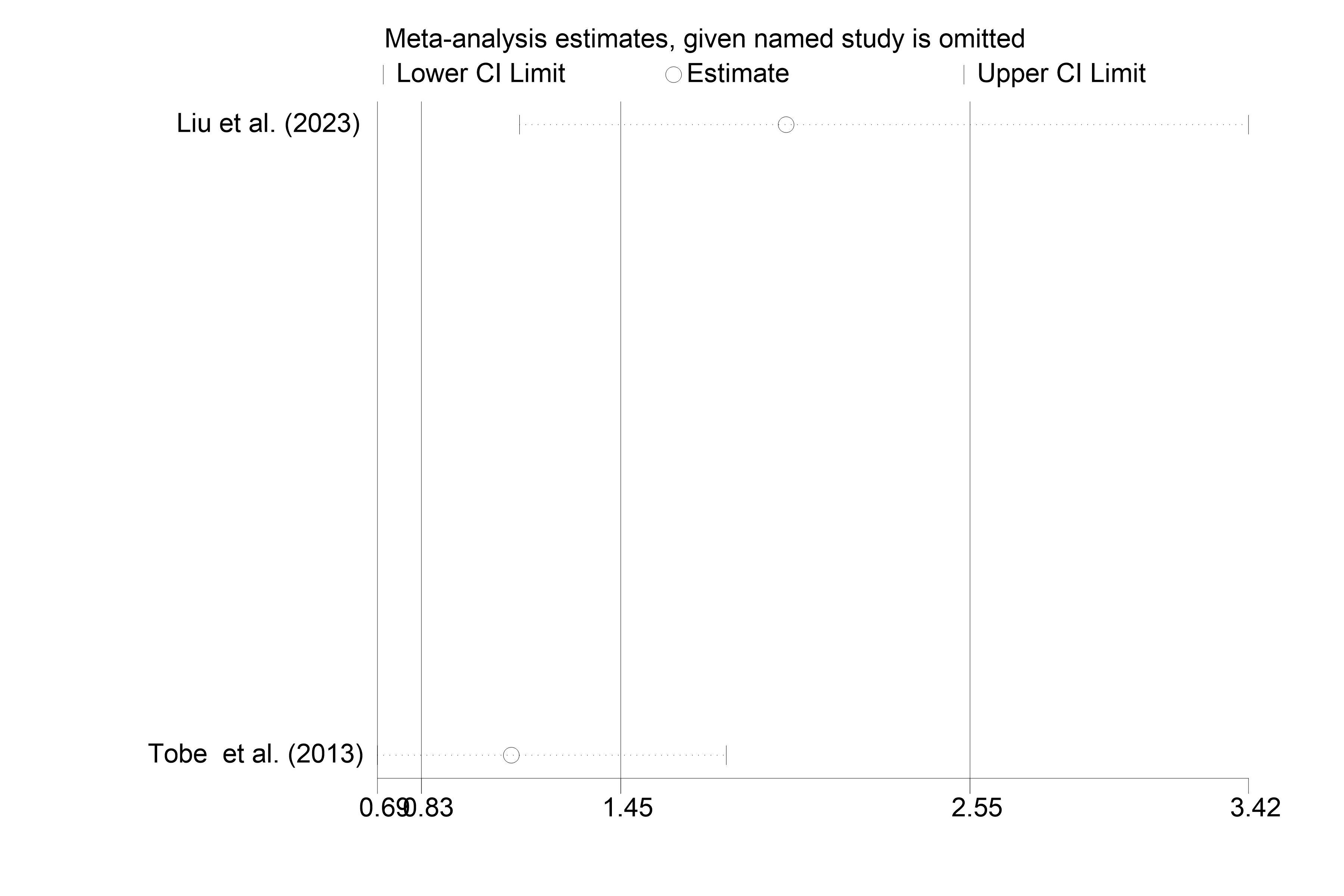


FigureS16. Sensitivity analysis for insurance,2025.


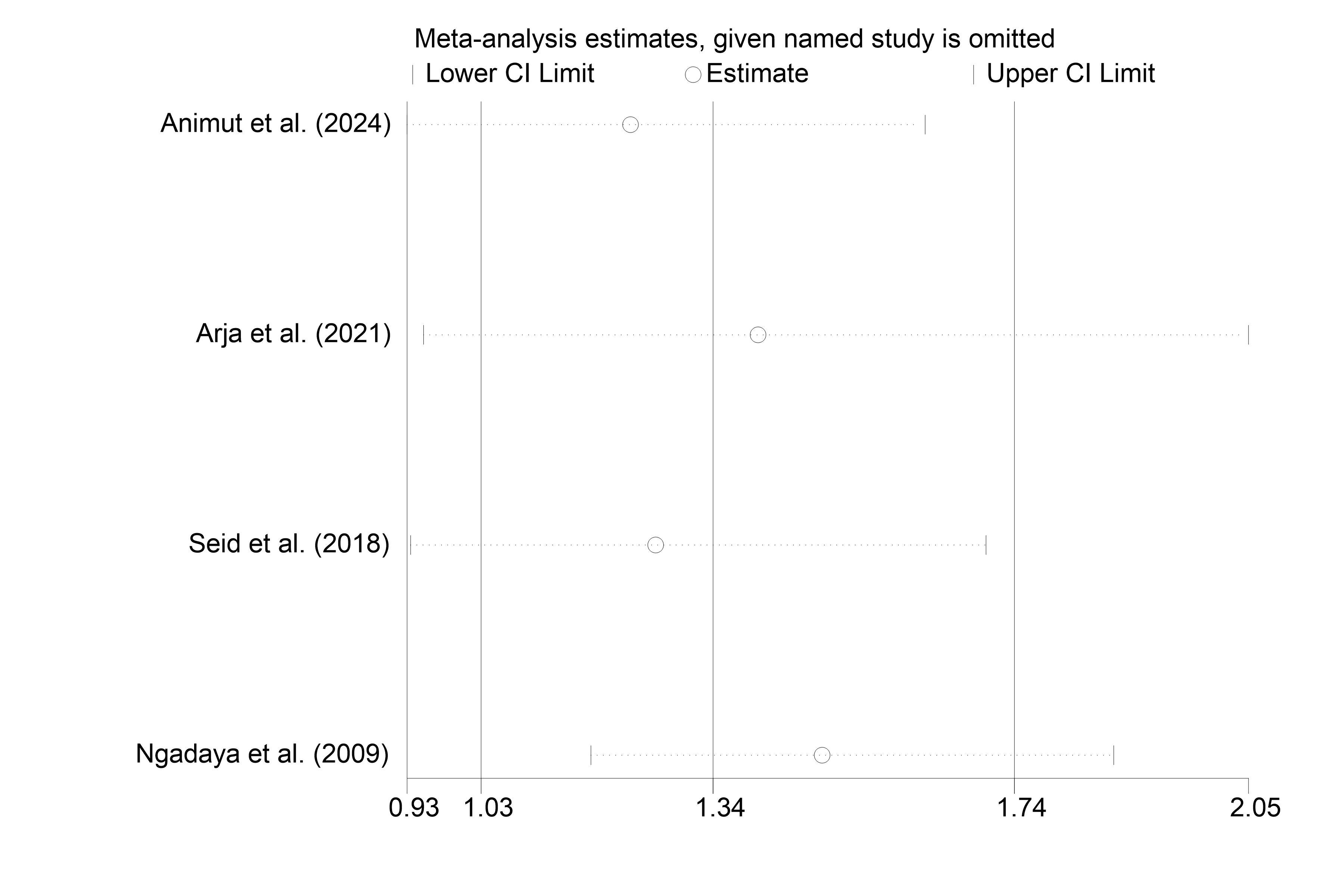


FigureS17. Sensitivity analysis for time of arrival at health services,2025.


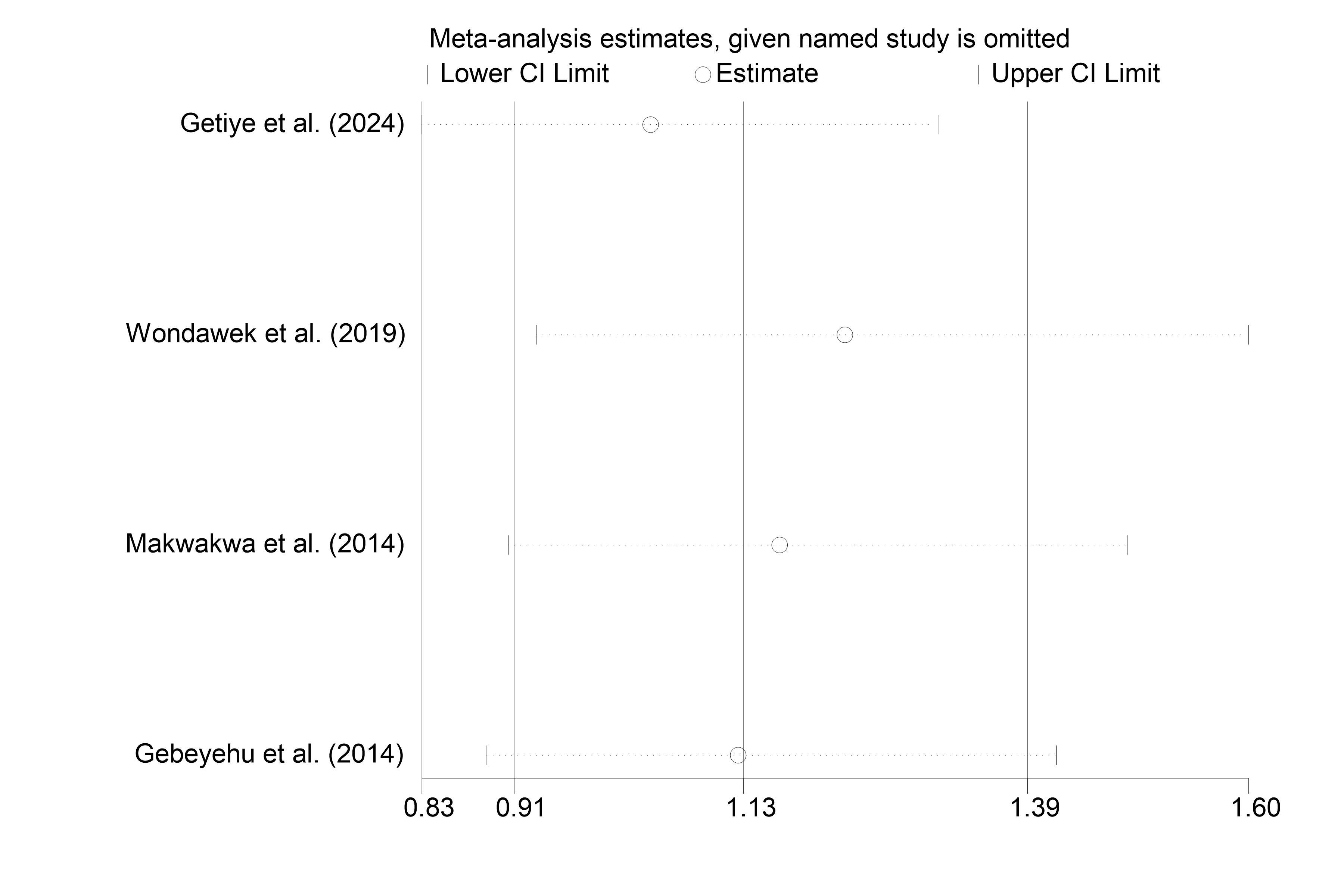


FigureS18. Sensitivity analysis for distance to health services,2025.
